# Effectiveness of different therapeutic measures combined with aerobic exercise as an intervention in patients with depression: a systematic review and network meta-analysis

**DOI:** 10.3389/fpsyt.2025.1573557

**Published:** 2025-07-25

**Authors:** Lei Chen, Aichun Li, Junlai Zhou, Wenhao Chen, Yujia Kou

**Affiliations:** School of Physical Education, Hainan Normal University, Haikou, Hainan, China

**Keywords:** depression, aerobic exercise, combination therapy, network meta-analysis, systematic review

## Abstract

**Background:**

Aerobic exercise (AE), as a non-pharmacological therapy, is an effective supplement to conventional depression treatments. However, a comprehensive assessment of combined AE interventions remains insufficient. This study aims to systematically evaluate the intervention effects of different therapies combined with AE in depression patients via network meta-analysis (NMA).

**Methods:**

Following the PICOS framework, literature was searched in PubMed, Web of Science, Cochrane Library, Embase, Scopus, CNKI, Wanfang, and CBM for randomized controlled trials (RCTs) until June 2024. Independent screening and data extraction were conducted. NMA utilized Stata 15.0 and R 4.4.1, with bias risk assessed by the Cochrane Risk of Bias tool and evidence quality assessed by CINeMA.

**Results:**

A total of 37 RCTs involving 3,362 patients with depression were included, evaluating five combined AE interventions. Results based on the area under the cumulative ranking curve indicated: (1) Hamilton Depression Rating Scale (HAMD): Electroconvulsive therapy + AE (ECT+AE) > repetitive transcranial magnetic stimulation + AE (rTMS+AE) > Traditional Chinese Medicine + AE (TCM+AE) > Selective Serotonin Reuptake Inhibitors + AE (SSRI+AE) > Cognitive Behavioral Therapy + AE (CBT+AE) > Physical Therapy (PT) > Exercise (EX) > CBT > TCM > Control Treatment (CT); (2) Beck Depression Inventory (BDI): SSRI+AE > ECT+AE > CBT+AE > EX > CBT > CT > PT; (3) Self-rating Depression Scale (SDS): TCM+AE > CBT+AE > CT > CBT.

**Conclusions:**

Current evidence suggests that combined aerobic exercise interventions are superior to monotherapy in the treatment of depression. Of these, SSRI+AE is the most recommended combination, with strong RCT evidence and high-quality evidence ratings. For other AE combination therapies, further validation in large, high-quality trials is necessary in the future.

**Systematic review registration:**

https://www.crd.york.ac.uk/PROSPERO/, identifier CRD42024594873.

## 1 Introduction

Depression is a common mental disorder that affects the mental health of hundreds of millions of people worldwide. According to the World Health Organization (WHO), depression has become one of the leading causes of disability in the world, severely affecting patients’ mood, cognition, and physiological functioning, and may even lead to an increase in suicidal behavior (1). With in-depth research on the pathophysiological mechanisms of depression, a variety of therapeutic modalities have been applied in clinical practice, including medication, psychotherapy, and physical therapy. However, these treatment modalities often face the limitations of poor compliance, many side effects, and high costs (2).

Exercise intervention, a non-pharmacological approach to depression management, has garnered significant attention owing to its cost-effectiveness, minimal side effects, and practical applicability (3). Research indicates that exercise exerts beneficial effects on individuals with depression through multiple mechanisms, such as elevating neurochemical levels, suppressing inflammatory responses, regulating the neuroendocrine system, and enhancing neuroplasticity (4). Network meta-analyses have also been used to compare the efficacy of different types of exercise on depression, showing that walking, jogging, yoga, and strength training are more effective than other exercises and that various types of exercise affect cognitive performance, back pain, and blood pressure (5).

Aerobic exercise (AE) refers to physical activity that improves oxygen utilization efficiency while fostering holistic physical and psychological health (6). Recent studies have established the therapeutic potential of aerobic exercise in alleviating depressive symptoms (7, 8). Evidence suggests that aerobic exercise ameliorates depressive symptoms through neurobiological modulation, including hippocampal upregulation of brain-derived neurotrophic factor (BDNF) levels. A regimen of moderate-intensity aerobic exercise (3-5 sessions weekly,≥30 minutes per session, sustained for 6-8 weeks) significantly reduces depressive symptoms in affected individuals (9). Growing experimental evidence supports the integration of aerobic exercise with other therapies to achieve synergistic benefits, thereby optimizing treatment efficacy and complementing conventional interventions (10–12).

Clinical guidelines from the United States, Australia, and New Zealand uniformly advocate integrating aerobic exercise into depression treatment protocols (13). The American Psychiatric Association endorses any aerobic exercise and resistance training as adjunct therapies, whereas Australian and New Zealand guidelines recommend ≥2-3 weekly high-intensity aerobic sessions combined with resistance training (14). However, existing research inadequately explores combination therapies involving aerobic exercise and fails to identify optimal integrated modalities for maximal therapeutic benefit. Furthermore, prior meta-analyses predominantly assessed aerobic exercise as a monotherapy (15–17), with limited investigation into its efficacy when combined with other treatments.

Therefore, this study used NMA to investigate the intervention effects of different treatment measures combined with aerobic exercise on patients with depression, with a view to identifying the most effective of these combined interventions, and to provide an evidence-based basis for treatment choices and nursing practice in depression.

## 2 Materials and methods

This study strictly adhered to the Priority Reporting Entries for Systematic Reviews and Meta-Analyses: a PRISMA Statement (18) and has been registered with the International Platform for the Registration of Systematic Reviews (PROSPERO) (registration number: CRD42024594873)^1^.

### 2.1 Data sources and search strategy

Based on the independent double-blind principle, two researchers followed the PICOS framework (19) and conducted computerized searches in PubMed, Web of Science, Cochrane Library, Embase, Scopus, CNKI, Wanfang Database, and China Biomedical Database. The researchers combined the corresponding subject terms and free text terms for each database, and the search timeframe was from the date of each database’s creation to June 6, 2024. The subject terms used in the literature search included “Exercise” AND “Combined Modality Therapy” AND “Depression” and “Randomized Controlled Trial”. Please refer to the **Supplementary Material** for details of the search strategy.

### 2.2 Selection and exclusion criteria

Inclusion criteria (1): Participants were patients with a confirmed diagnosis of depression according to the Diagnostic and Statistical Manual of Mental Disorders (DSM) (20), International Classification of Diseases (ICD) (21), or Chinese Classification of Mental Disorders (CCMD) (22), or patients with depression level scores above the threshold as determined by other clinical diagnostic methods and commonly used depression assessment scales (23–25). Comorbidity was not used as an exclusion criterion (2). The control group received conventional treatment, including medication, psychotherapy, physical therapy, or single-mode exercise therapy. The experimental group combines aerobic exercise (such as walking, jogging, dancing, etc.) with low to moderate intensity on a continuous basis, with the treatment of the control group (3). The results of this study were measured based on the severity of depressive symptoms, which were assessed using the HAMD, BDI, and SDS scores (4). The study type was RCT.

Exclusion criteria (1): studies in which diagnostic criteria were not clearly described (2); studies in which a single treatment measure was used in the test group (3); studies in which the full text was not available; and (4) studies in which information was incomplete and data could not be extracted.

### 2.3 Literature screening and data extraction

Two researchers conducted the inclusion and screening of the literature in an independent double-blind manner and carried out cross-checking. In case of any discrepancies during the inclusion process, a third researcher would be involved to make the final decision. Finally, literature management was carried out using EndNoteX9 software, and data were extracted step by step in accordance with a pre-designed table, including the first author of the literature, the title, the year of publication, the basic information of the research subjects, the interventions taken, and the main outcome measures.

### 2.4 Risk of bias evaluation of included studies

Based on the recommended risk of bias assessment tool in the Cochrane Handbook 5.1.0, the RevMan 5.4 software was used to evaluate the risk of bias in RCTs according to six aspects: random sequence generation, allocation concealment, blinding of participants and personnel, completeness of outcome data, selective reporting, and other bias (26). When all items were assessed as “low risk,” the study was classified as grade A. If some items were assessed as “low risk,” it was classified as grade B. If none were assessed as “low risk,” it was considered grade C (27).

### 2.5 Statistical analysis

In this study, NMA was performed using Stata 15.0 software. For the closed-loop structure in the network evidence graph, the node-split method was applied to test for consistency; a consistency model was used if P > 0.05, and an inconsistency model was used otherwise. Intervention ranking was assessed using the surface under the cumulative ranking curve (SUCRA; 0% to 100%, higher values indicate better efficacy). Publication bias was assessed using Egger’s test and funnel plots. Heterogeneity was evaluated using τ² and its p-value for qualitative assessment, alongside I² for quantitative estimation (I² < 25%: low; 25–50%: moderate; > 50%: substantial; > 75%: high heterogeneity). To address substantial heterogeneity (I² > 50%), NMA regression analyses were conducted using the R software gemtc package to explore potential sources (e.g., publication year, age, diagnostic tools, baseline symptoms, intervention duration, and frequency). Finally, the CINeMA framework was applied to assess the risk of bias and evidence quality (28).”

## 3 Results

### 3.1 Results of the literature search

**Figure 1:**
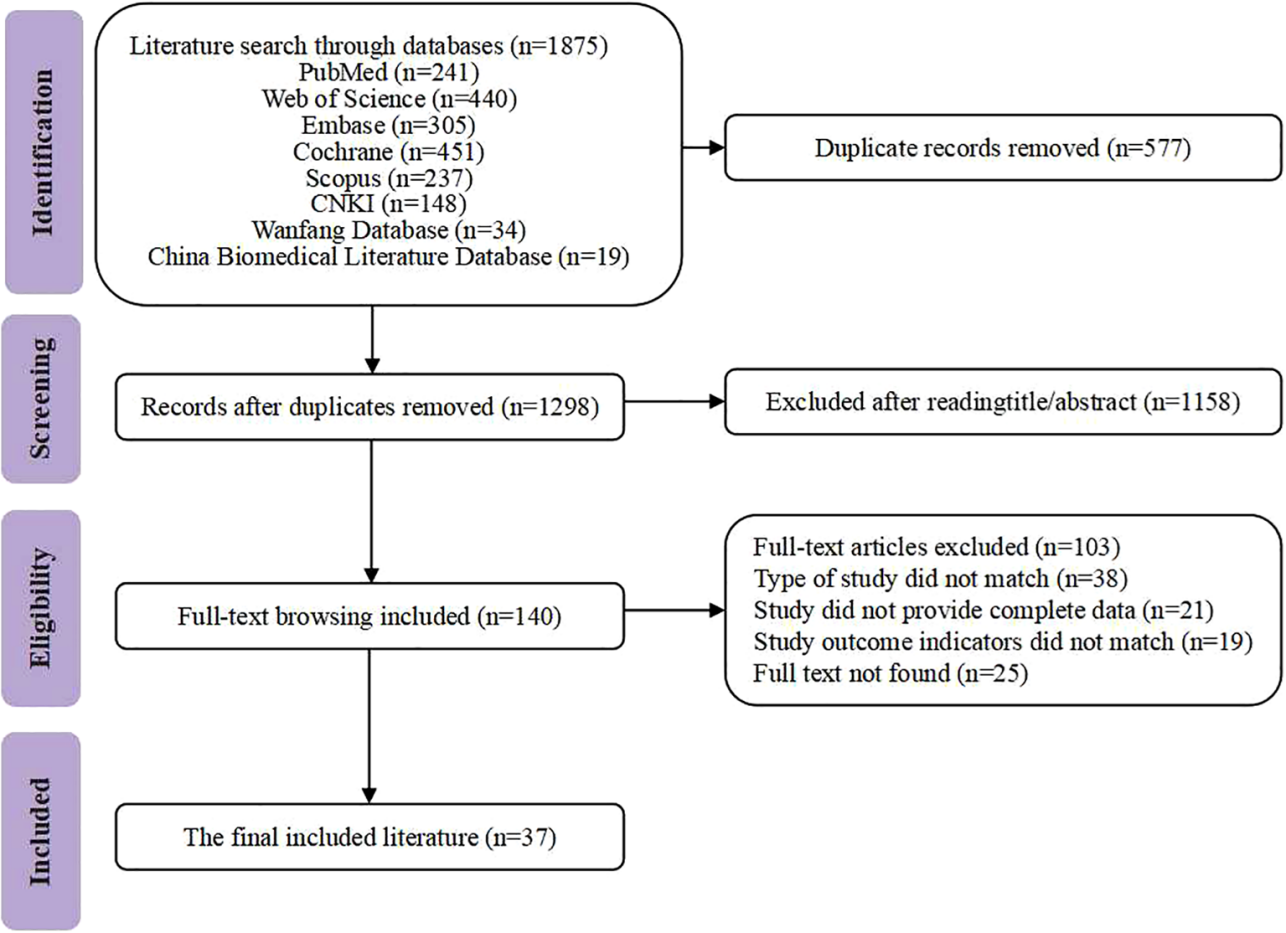
Flowchart of literature screening.

A comprehensive search of 8 databases yielded 1,875 articles relevant to the research topic. After merging and filtering with EndNote X9, 577 duplicates were removed. Then, 140 articles were preliminarily selected based on titles and abstracts. Finally, 37 RCTs were included after full-text reviews of the 140 articles. **Figure 1** shows the literature screening process.

### 3.2 The characteristics of studies

This study included a total of 37 articles (29–65), encompassing 3,362 patients with depression and evaluating five distinct combined aerobic exercise interventions. Among these, 19 studies investigated selective serotonin reuptake inhibitors combined with aerobic exercise (SSRI+AE), 12 studies examined cognitive behavioral therapy combined with aerobic exercise (CBT+AE), 4 studies focused on Traditional Chinese Medicine combined with aerobic exercise (TCM+AE), 2 studies assessed repetitive transcranial magnetic stimulation combined with aerobic exercise (rTMS+AE), and 1 study explored electroconvulsive therapy combined with aerobic exercise (ECT+AE). The baseline characteristics of the included studies are presented in **Table 1**.

**Table 1:** Basic characteristics of the included studies.

Study	Year	Country	Sample size		Diagnostic standards	Intervention		Intervention period	Outcome measures
			E	C		E	C		
Wang (29)	2021	China	30	30	CCMD-3	SSRI+AE	CT	8week	①
Siqueira (30)	2016	Canada	29	28	DSM-IV	SSRI+AE	CT	4week	①②
Legrand (31)	2016	France	T1:14 T2:10	10	DSM-IV	T1:SSRI+AE T2:EX	CT	lasts 10 days	②
Carneiro (32)	2015	Portugal	9	10	ICD-10	SSRI+AE	CT	16week	②
Murri (33)	2015	Italy	42	42	DSM-IV	SSRI+AE	CT	24week	①
Mota-Pereira (34)	2011	Portugal	10	19	DSM-IV	SSRI+AE	CT	12week	①②
Cerda (35)	2011	Philippines	41	41	ICD-10	SSRI+AE	CT	8week	②
Herman (36)	2002	USA	T1:44 T2:39	41	DSM-IV	T1:SSRI+AE T2:EX	CT	16week	①②
Tan LY (37)	2023	China	60	60	CCMD-3	SSRI+AE	CT	12week	①
Fu ZJ (38)	2022	China	45	45	CGPD-2	SSRI+AE	CT	8week	①
Xu RZ (39)	2020	China	70	70	ICD-10	SSRI+AE	CT	6week	①
Cai J (40)	2020	China	45	37	DSM-V	SSRI+AE	CT	6week	①
Du Y (41)	2019	China	30	30	ICD-10	SSRI+AE	CT	6week	①
Ge CJ (42)	2018	China	41	41	ICD-10	SSRI+AE	CT	6week	①
Ning QF (43)	2018	China	68	66	DSM-V	SSRI+AE	CT	6week	①
Lu B (44)	2017	China	41	39	clinical diagnosis	SSRI+AE	CT	12week	①
Wang L (45)	2016	China	39	36	DSM-IV	SSRI+AE	CT	24week	①
Huang J (46)	2013	China	30	30	HAMD≥8	SSRI+AE	CT	24week	①
									①
Wu YL (47)	2023	China	42	42	HAMD≥7	TCM+AE	TCM	12week	①
Mi JG (48)	2021	China	39	38	DSM-IV	TCM+AE	CT	6week	①③
Chen LY (49)	2020	China	29	29	CCMD-3	TCM+AE	CT	12week	①③
Song ZL (50)	2019	China	48	48	CCMD-3	TCM+AE	CBT	12week	①
Saha (51)	2024	USA	T1:17 T2:21 T3:17	20	DSM-IV	T1:CBT+AE T2:EX T3:CBT	CT	12months	②
Bieber (52)	2021	Germany	47	36	DSM-IV	CBT+AE	CBT	3months	②
Groot (53)	2019	Athens	T1:25 T2:30 T3:24	28	DSM-IV	T1:CBT+AE T2:EX T3:CBT	CT	12week	②
Kerling (54)	2015	Germany	22	20	DSM-IV	CBT+AE	CBT	6week	②
Piette (55)	2011	USA	145	146	PHQ-9	CBT+AE	CT	12months	②
Wang L (56)	2021	China	31	30	ICD-10	CBT+AE	CBT	4week	①
Xu RZ (57)	2021	China	46	45	ICD-10	CBT+AE	CBT	12week	①③
Han L (58)	2020	China	42	42	ICD	CBT+AE	CBT	8week	①③
Wu Y (59)	2020	China	46	46	SDS ≥50 and HAMD >7	CBT+AE	CT	24week	①③
Wu XB (60)	2018	China	35	35	HAMD score	CBT+AE	CBT	6week	①
Qin SH (61)	2018	China	35	35	HAMD score	CBT+AE	CBT	6week	①
Li HW (62)	2014	China	25	24	CCMD-3	CBT+AE	CBT	24week	①③
Fang M (63)	2021	China	80	80	ICD-10	rTMS+AE	PT	4week	①
Zhu BY (64)	2019	China	150	150	HAMD≥20	rTMS+AE	PT	4week	①
Salehi (65)	2016	Iran	T1:20 T2:20	20	DSM-IV	T1:ECT+AE T2:EX	PT	4week	①②

E, experiment group; C, control group; T1-T3, represent different groups in the same trial; SSRI+AE, selective serotonin reuptake inhibitors + aerobic exercise; ECT+AE, electroconvulsive therapy + aerobic exercise; TCM+AE, traditional Chinese medicine + aerobic exercise; CBT+AE, cognitive behavioral therapy + aerobic exercise; rTMS+AE, repetitive transcranial magnetic stimulation + aerobic exercise; CT, conventional treatment; EX, exercise therapy; PT, physical therapy; CBT, cognitive behavioral therapy; TCM, traditional Chinese medicine treatment; CCMD-3, Chinese Classification and Diagnostic Criteria of Mental Disorders (3rd Edition); DSM-IV, Diagnostic and Statistical Manual of Mental Disorders (4th Edition); CGPD-2, Chinese Guidelines for the Prevention and Treatment of Depressive Disorders (2nd Edition); ICD-10, International Classification of Diseases and Health-Related Problems, 10th Revision; NICE-CG28, National Institute for Health and Clinical Excellence Clinical Guideline 28 (UK); PHQ-9, Patient Health Questionnaire-9; ① Hamilton Depression Rating Scale score; ② Beck Depression Inventory score; ③ Zung Self-Rating Depression Scale score.

### 3.3 Quality assessment

**Figure 2:**
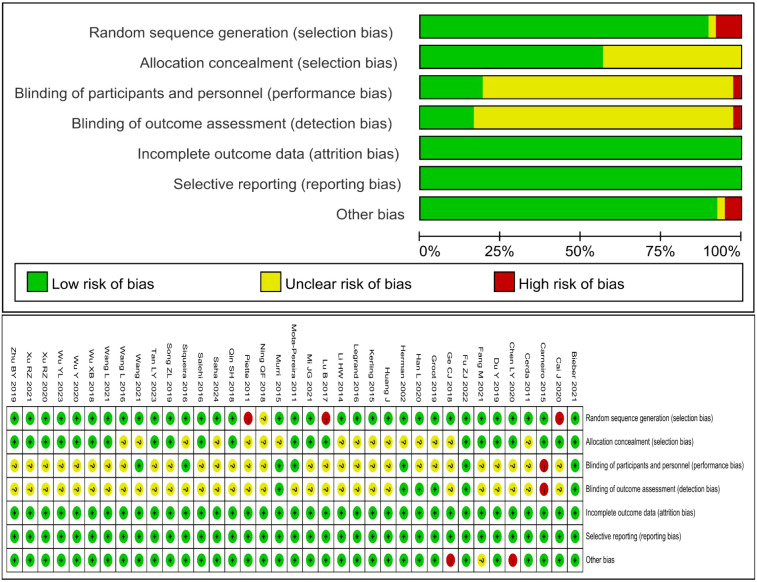
Quality assessment of included studies.

The quality of the included literature was evaluated; 2 were graded A (38, 52) and 35 were graded B (29–37, 39–51, 53–65). The quality of the included literature was assessed by a quality assessment. Of these, 35 used randomization sequences, 24 implemented allocation method concealment, 4 were blinded to both subjects and assessors, and all of the included literature reported on the primary outcome indicators. Detailed results are shown in **Figure 2**.

### 3.4 Results of reticulated meta-analysis

#### 3.4.1 Web evidence map

**Figure 3:**
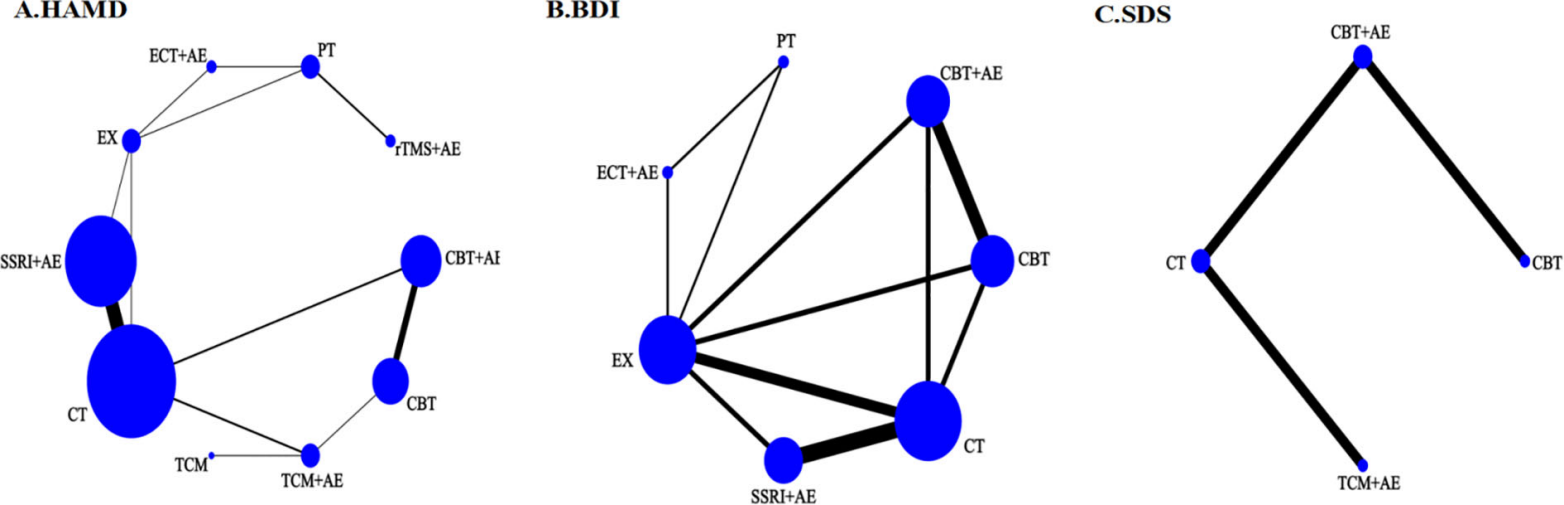
Network structure of different treatment measures. **(A)** Network structure diagram of HAMD outcomes; **(B)** Network structure diagram of BDI outcomes; **(C)** Network structure diagram of SDS outcomes.

The HAMD score, BDI score, and SDS score were used to compare the intervention effects of different interventions, and their network relationships are shown in **Figure 3**. The dots in the network evidence map represent the interventions for treating depression. The size of the dots reflects the sample size of the intervention, while the thickness of the lines between the dots indicates the number of related studies. The results demonstrated that SSRI+AE had the highest number of supporting studies compared to other interventions.

#### 3.4.2 Inconsistency check

Inconsistency tests were performed on the closed loops formed by HAMD scores and BDI scores. The results showed that the inconsistency factor (IF) values of some closed loops were higher than 1, but the 95% CIs of all closed loops included 0, indicating no inconsistency in outcome indicators (see **Table 2**). Furthermore, the node-splitting method was used to perform global consistency and local consistency tests. All results showed P > 0.05, suggesting good global and local consistency. Therefore, the consistency model was adopted for the network meta-analysis in this study.

**Table 2:** Results of the inconsistency test for the closed loop.

Outcome measures	Ring of closure	IF	95%CI
HAMD	CT-EX-SSRI+AE	3.57	0.00-14.02
	CBT-CBT+AE-CT-TCM+AE	0.65	0.00-626
BDI	CBT-CBT+AE-CT	2.459	0.00-8.84
	CBT-CBT+AE-EX	2.413	0.00-6.93
	CBT+AE-CT-EX	1.381	0.00-9.83
	CBT-CT-EX	1.325	0.00-8.43
	CT-EX-SSRI+AE	1.183	0.00-13.51

#### 3.4.3 Comparative results for key indicators

**Figure 4:**
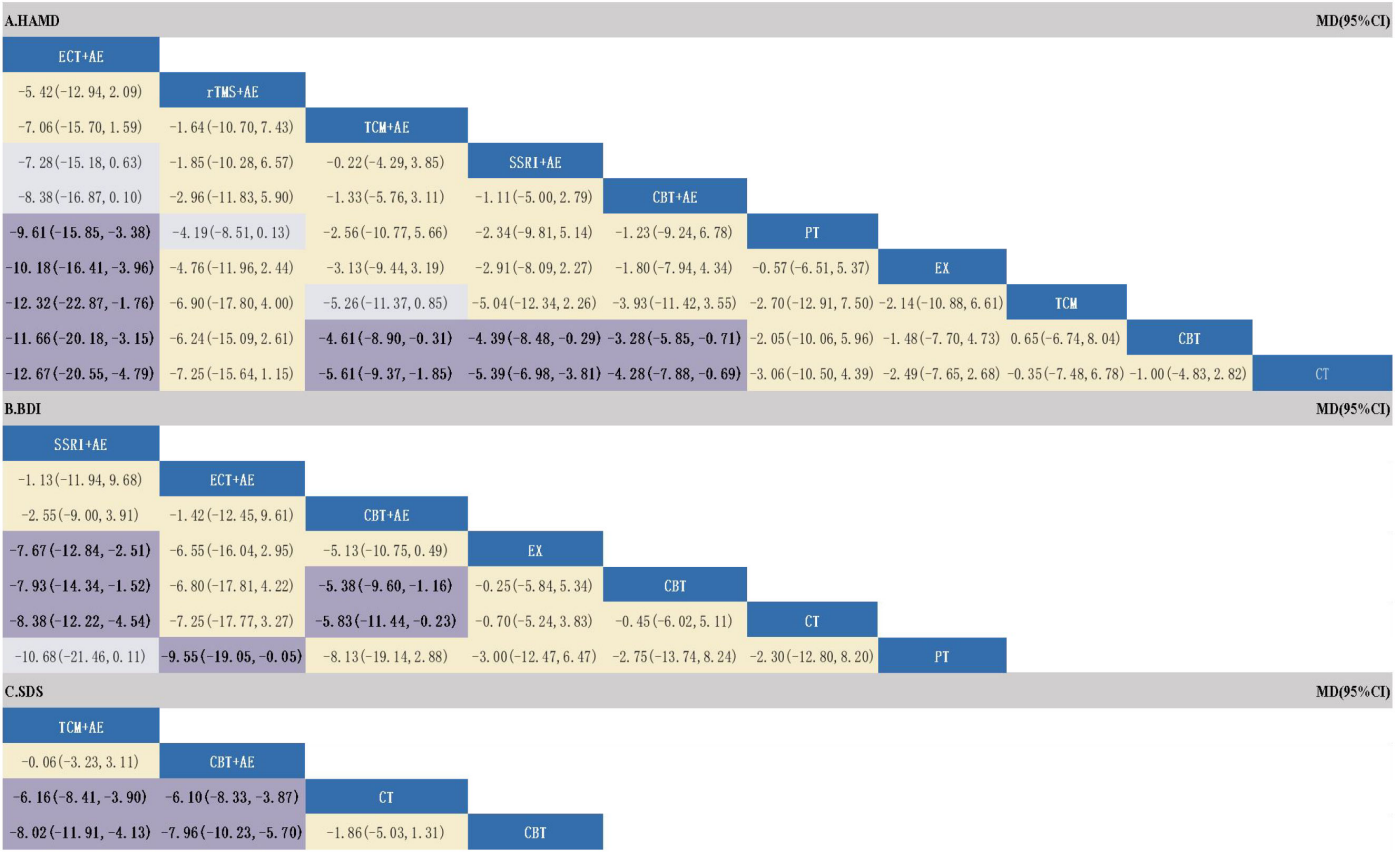
Results of a network meta-analysis of different treatment measures (MD95%CI). **(A)** ranking of different combinations for HAMD outcomes; **(B)** ranking of different combination treatments for BDI outcomes; **(C)** ranking of different combination treatments for SDS outcomes.

Twenty-nine RCTs reported HAMD scores, involving 5 combined treatment measures. The results of NMA showed (**Figure 4A**) that compared with conventional treatment, ECT+AE (MD: -9.61, 95%CI: -15.85 to -3.38), TCM+AE (MD: -5.61, 95%CI: -9.37 to -1.85), SSRI+AE (MD: -5.39, 95%CI: -6.89 to -3.81), and CBT+AE (MD: -4.28, 95%CI: -7.88 to -0.69) were more effective. Compared with single CBT treatment, TCM+AE (MD: -4.16, 95%CI: -5.51 to -2.81), SSRI+AE (MD: -4.16, 95%CI: -5.51 to -2.81), and CBT+AE (MD: -4.16, 95%CI: -5.51 to -2.81) were more effective. It is worth noting that ECT+AE was superior to all single treatment measures.

**Figure 5:**
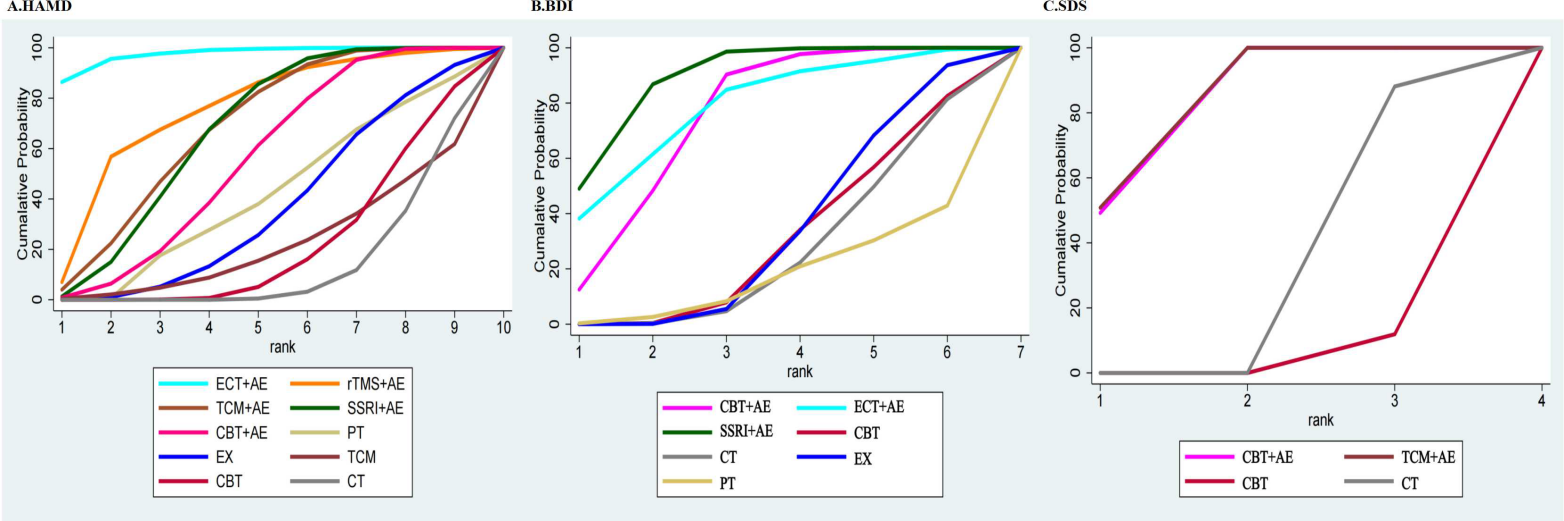
**(A)** SUCRA for HAMD outcomes; **(B)** SUCRA for BDI outcomes; **(C)** SUCRA for SDS outcomes.

The SUCRA rankings of each treatment measure were ECT+AE (97.6%) > rTMS+AE (75.5%) > TCM+AE (68.4%) > SSRI+AE (67.3%) > CBT+AE (55.6%) > Physical Therapy (41.2%) > Exercise Therapy (36.5%) > CBT (22.1%) > TCM (22%) > CT (13.7%). However, it should be particularly noted that the top two ranked ECT+AE and rTMS+AE were contributed by only 1 and 2 studies, respectively. Although their SUCRA values were high, the evidence base was very weak, and interpretation should be extremely cautious. The cumulative probability comparison is shown in **Figure 5A**, and the detailed SUCRA ranking probabilities are shown in **Supplementary Table 11**.

#### 3.4.4 Comparative results for secondary indicators

Twelve RCTs reported BDI scores, involving 3 combined treatment measures. The NMA results showed (**Figure 4B**) that SSRI+AE was superior to conventional treatment (MD: -8.38, 95%CI: -12.22 to -4.54), single EX (MD: -7.67, 95%CI: -12.84 to -2.51), and single CBT (MD: -7.93, 95%CI: -14.34 to -1.52). CBT+AE was superior to conventional treatment (MD: -5.83, 95%CI: -11.44 to -0.23) and single CBT (MD: -5.38, 95%CI: -9.60 to -1.16). ECT+AE was only superior to single PT (MD: -9.55, 95%CI: -19.05 to -0.05). The SUCRA rankings were SSRI+AE (88.8%) > ECT+AE (78.1%) > CBT+AE (75%) > EX (33.1%) > CBT (30.9%) > CT (26.6%) > PT (17.3%). The cumulative probability comparison is shown in **Figure 5B**, and the detailed SUCRA ranking probabilities are presented in **Supplementary Table 12**.

Six RCTs reported SDS scores, involving 2 combined treatment measures. The NMA results showed (**Figure 4C**) that TCM+AE was superior to conventional treatment (MD: -6.16, 95%CI: -8.41 to -3.90) and single CBT (MD: -8.02, 95%CI: -11.91 to -4.13). CBT+AE was also superior to conventional treatment (MD: -6.10, 95%CI: -8.33 to -3.87) and single CBT (MD: -7.96, 95%CI: -10.31 to -5.70). The SUCRA rankings were TCM+AE (83.6%) > CBT+AE (83.1%) > CT (29.4%) > CBT (4%). The cumulative probability comparison is shown in **Figure 5C**, and the detailed SUCRA ranking probabilities are presented in **Supplementary Table 13**.

#### 3.4.5 Heterogeneity and regression analysis

Heterogeneity was assessed using τ², I², and Q statistics, indicating substantial heterogeneity across studies (**Supplementary Table 14**). We therefore conducted meta-regression to examine potential moderators: mean age, publication year, diagnostic criteria, baseline symptom severity, session duration, intervention frequency, and treatment duration. No significant moderating effects were identified for any covariate (**Supplementary Table 15**), indicating that the substantial heterogeneity persisted, but the main comparative findings of the NMA were not substantially altered by these factors.

#### 3.4.6 Publication bias

**Figure 6:**
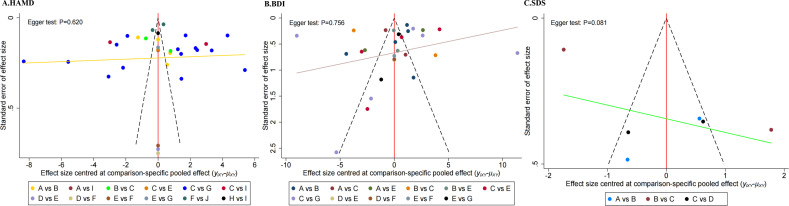
Funnel plot of publication bias for different outcome indicators. **(A)** Comparison-adjusted funnel plot for HAMD outcomes; **(B)** Comparison-adjusted funnel plot for BDI outcomes; **(C)** Comparison-adjusted funnel plot for SDS outcomes. A = CBT+AE; B = CT; C = ECT+AE; D = EX; E = SSRI+AE; F = TCM+AE; G = rTMS+AE.

The comparison-adjusted funnel plots for HAMD, BDI, and SDS scores showed asymmetrical scatter distribution, with some points outside the funnel, implying potential publication bias or small-sample effects. However, Egger’s test showed no significant publication bias (P > 0.05). See **Figure 6**.

#### 3.4.7 Quality of evidence evaluation

According to the CINeMA evidence quality assessment results (**Supplementary Table 16**), only SSRI+AE was rated as having high confidence, while most of the other evidence had “low” confidence and a small portion had “very low” confidence. The reasons for this were significant limitations due to bias risk and imprecision. However, the directness of the research evidence and the low likelihood of publication bias provided assurance for the reliability of the study results.

## 4 Discussion

Depression is a mental disorder that significantly impacts the quality of life, with core symptoms including persistent low mood, diminished interest, and anhedonia (66). The variability in severity and clinical manifestations of depression poses challenges for treatment, and conventional monotherapies often yield suboptimal outcomes (67). Exercise therapy, particularly aerobic exercise, has emerged as a vital complementary approach to depression treatment due to its minimal side effects, cost-effectiveness, and ease of implementation (68). This study synthesized 37 RCTs from eight databases through a network meta-analysis. Pairwise comparisons of interventions combining aerobic exercise revealed that five therapeutic approaches (SSRI, TCM, rTMS, CBT, ECT) combined with aerobic exercise outperformed conventional monotherapies.

The NMA based on HAMD scores indicated that physical therapies combined with aerobic exercise (ECT+AE and rTMS+AE) demonstrated significant potential advantages in improving depressive symptoms, with SUCRA values reaching 97.6% and 75.5%, respectively. Mechanistically, ECT modulates neurotransmitter equilibrium and neural circuit reorganization in the cerebral cortex via electrical stimulation, restoring homeostasis in the excitatory-inhibitory system and thereby rapidly alleviating core depressive symptoms (69). As a non-invasive physical intervention, rTMS utilizes magnetic pulses to target and regulate neuronal activity in the dorsolateral prefrontal cortex, promoting neuroplasticity and enhancing emotional regulation (70). Aerobic exercise facilitates endorphin release, elevates BDNF levels, and concurrently improves cerebral perfusion and mitigates oxidative stress, creating a multimodal intervention targeting both neurobiological and psychological-behavioral pathways. Its synergistic effects with the aforementioned physical therapies may further enhance therapeutic outcomes (71). However, the current evidence base exhibits significant limitations: only one RCT supports the efficacy of ECT+AE, and only two RCTs support rTMS+AE. This extremely limited number of studies substantially increases the risk of small-study bias and overestimation of effect sizes. Furthermore, CINeMA evidence quality assessment rated their reliability lower than interventions supported by multiple studies (SSRI+AE). Consequently, although the SUCRA ranking suggests potential clinical promise for these combination physical therapies, the conclusion must be regarded as highly exploratory due to the fragility of the evidence. Large-scale, multi-center clinical trials are urgently required to validate their long-term efficacy and safety.

In interventions combining pharmacotherapy with aerobic exercise, TCM+AE showed a marginally higher SUCRA ranking than SSRI+AE, with SUCRA values of 68.9% and 67.1%, respectively. This marginal difference may suggest a potential advantage for TCM+AE, whose mechanism emphasizes personalized holistic regulation, potentially enhancing efficacy by improving the synergistic mind-body state (72). In contrast, SSRIs primarily improve mood by increasing synaptic serotonin concentrations but are limited by delayed onset and insufficient response in some patients (73). Aerobic exercise further amplifies the synergistic effects of both interventions by promoting neurotrophic factor release, enhancing neuroplasticity, and modulating oxidative stress-inflammatory pathways (74). However, it is crucial to emphasize caution: evidence supporting the efficacy of TCM+AE originates from only four RCTs, and its CINeMA evidence quality rating is lower than that of SSRI+AE due to insufficient sample sizes and methodological heterogeneity. This disparity in evidence quality may significantly diminish the clinical interpretability of the SUCRA ranking difference and even carries a risk of small-study bias overestimating the effect. Therefore, although TCM+AE shows potential based on theoretical mechanisms and preliminary data, the findings are insufficient to confirm its significant clinical superiority over SSRI+AE. It should rather be regarded as a potential optimization strategy requiring validation in large-scale, high-quality studies. Future research also urgently needs more rigorously designed clinical trials specifically investigating the synergistic mechanisms between TCM and exercise therapy.

Cognitive behavioral therapy combined with aerobic exercise (CBT+AE) demonstrated superior efficacy to monotherapy in SUCRA rankings across three depression scales, yet underperformed relative to other combined exercise interventions. This discrepancy may stem from inherent tensions between CBT+AE’s therapeutic characteristics and conventional assessment frameworks. As an individualized psychological intervention, CBT+AE’s effectiveness depends critically on therapist-patient engagement and requires sustained behavioral-cognitive restructuring, a process that typically necessitates an extended duration for stable therapeutic effects to manifest (75, 76). While aerobic exercise provides rapid mood improvement through endorphin release and neuroplasticity modulation, fluctuations in patient adherence to psychological interventions may compromise synergistic benefits (77). Notably, existing depression scales primarily capture somatic symptom improvement rather than psychological mechanisms targeted by CBT+AE, such as cognitive restructuring or behavioral activation (78). This measurement misalignment potentially obscures CBT+AE’s long-term advantages in short-term evaluations. Promisingly, emerging evidence suggests dose-response efficacy for CBT+AE in comorbid conditions, for example, OCD, and group-based exercise formats may enhance treatment adherence through social interaction (79). Future protocols should integrate motivational enhancement strategies and multidimensional assessment tools to fully realize this intervention’s clinical potential.

This study has the following limitations. First, although both Chinese and English literature were systematically searched and included, the absence of studies in other languages may compromise the comprehensiveness of the evidence, potentially omitting key findings from specific regions or cultural contexts. Second, significant clinical heterogeneity was present. Although a random-effects model was applied for adjustment and regression analyses were conducted, variations in treatment response across different populations could still confound the efficacy assessment. Third, the outcomes overly relied on depression scale scores, lacking multidimensional assessments such as patient-reported experiences, quality of life, and functional recovery, making it difficult to fully capture the interventions’ actual clinical value. Crucially, the number of studies investigating physical therapy combinations (only 1 for ECT+AE and 2 for rTMS+AE) and TCM combinations (only 4 for TCM+AE) was severely limited. This not only resulted in lower CINeMA evidence quality ratings but also introduced a risk of small-study bias in the SUCRA rankings, highlighting considerable uncertainty in the results. Additionally, the overall quality of the included studies was suboptimal, primarily due to inadequate allocation concealment and double-blinding, which constrained the robustness of the conclusions. Therefore, future research urgently needs to address these evidence gaps through standardized intervention protocols, multidimensional outcome assessments, and large-scale trials.

## 5 Conclusion

Based on the current evidence, aerobic exercise combination therapies demonstrate superior efficacy over monotherapy. Among these, SSRI+AE emerges as the most robustly supported intervention, with the highest volume of RCT evidence and CINeMA quality rating. Although ECT+AE and rTMS+AE show good SUCRA rankings, they have a limited number of studies and can only be used as exploratory findings. TCM+AE and CBT+AE exhibit potential but are constrained by methodological heterogeneity and assessment limitations. Future research should prioritize large multicenter trials with standardized protocols and multidimensional outcomes to evaluate physical therapy combinations long-term, alongside mechanistic studies on TCM-exercise synergy for precision therapy.

## Data Availability

The original contributions presented in the study are included in the article/**Supplementary Material**. Further inquiries can be directed to the corresponding author.

## Footnotes

1 https://www.crd.york.ac.uk/PROSPERO/
