# Supplementary material for: Effectiveness of different therapeutic measures combined with aerobic exercise as an intervention in patients with depression: a systematic review and network meta-analysis
Source: Front Psychiatry. 2025 Jul 25;16:1573557. doi: 10.3389/fpsyt.2025.1573557 (PMC12332749; doi:10.3389/fpsyt.2025.1573557)
Supplement: Supplementary file 1 [file DataSheet1.pdf]

Supplementary Material

CONTENT

1. Supplementary Table **.......... 2**

Table S1 PRISMA NMA Checklist of Items to Include When Reporting A Systematic Review Involving a Network Meta-analysis 2

Table S2 Search strategy in PubMed 6

Table S3 Search strategy in Web of Science 6

Table S4 Search strategy in Cochrane Library 7

Table S5 Search strategy in Embase 7

Table S6 Search strategy in Scopus 8

Table S7 Search strategy in CNKI 8

Table S8 Search strategy in Wanfang database 8

Table S9 Search strategy in China Biomedical Literature Database 8

Table S10 Inclusion and Exclusion Criteria 9

Table S11 SUCRA cumulative probability ranking (based on HAMD) 9

Table S12 SUCRA cumulative probability ranking (based on BDI) 10

Table S13 SUCRA cumulative probability ranking (based on SDS) 10

Table S14 Evaluation of heterogeneity 10

Table S15 Outcome of Network Meta-Regression (median and 95%Crl) 10

Table S16 The Overall Certainty of Evidence (CINeMA，Direct) 11

Table S17 The Overall Certainty of Evidence (CINeMA，Indirect) 11

2．Supplementary Figure **.......... 14**

Figure S1 | Meta-analysis of the effect of different treatment measures combined with aerobic exercise on the intervention of depressed patients (based on HAMD) 14

Figure S2 | Meta-analysis of the effect of different treatment measures combined with aerobic exercise on the intervention of depressed patients (based on BDI) 15

Figure S3 | Meta-analysis of the effect of different treatment measures combined with aerobic exercise on the intervention of depressed patients (based on SDS) 16

Figure S4 | Network meta-analysis interval plots for different treatment measures (HAMD-based) 16

Figure S5 | Network meta-analysis interval plots for different treatment measures (BDI-based) 17

Figure S6 | Network meta-analysis interval plots for different treatment measures (SDS-based) 17

Figure S 7 | HAMD-based forest maps 18

Figure S8 | BDI-based forest maps 18

Figure S9 | Inconsistency test for closed loops (based on HAMD) 19

Figure S10 | Inconsistency test for closed loops (based on BDI) 19

Figure S11 | Contribution of the HAMD scale 20

Figure S12 | Contribution of the BDI scale 20

Figure S13 | Contribution of the SDS scale 21

Figure S14 | Summary of limitations of included studies. 21

1. *Supplementary Table*

Table S1 PRISMA NMA Checklist of Items to Include When Reporting A Systematic Review Involving a Network Meta-analysis

Section/Topic	Item #	Checklist Item	Reported on Page #
**TITLE**			
Title	1	Identify the report as a systematic review *incorporating a network meta-analysis (or related form of meta-analysis).*	***1***
**ABSTRACT**			
Structured summary	2	Provide a structured summary including, as applicable:<br>**Background:** main objectives<br>**Methods:** data sources; study eligibility criteria, participants, and interventions; study appraisal; and *synthesis methods, such as network meta-analysis.*<br>**Results:** number of studies and participants identified; summary estimates with corresponding confidence/credible intervals; *treatment rankings may also be discussed. Authors may choose to summarize pairwise comparisons against a chosen treatment included in their analyses for brevity.*<br>**Discussion/Conclusions:** limitations; conclusions and implications of findings.<br>**Other:** primary source of funding; systematic review registration number with registry name.	1-2
**INTRODUCTION**			
Rationale	3	Describe the rationale for the review in the context of what is already known*, including mention of why a network meta-analysis has been conducted.*_	***2 (Introduction)***
Objectives	4	Provide an explicit statement of questions being addressed, with reference to participants, interventions, comparisons, outcomes, and study design (PICOS).	2 (Abstract), 2 (Introduction)
**METHODS**			
Protocol and registration	5	Indicate whether a review protocol exists and if and where it can be accessed (e.g., Web address); and, if available, provide registration information, including registration number.	3
Eligibility criteria	6	Specify study characteristics (e.g., PICOS, length of follow-up) and report characteristics (e.g., years considered, language, publication status) used as criteria for eligibility, giving rationale. *Clearly describe eligible treatments included in the treatment network, and note whether any have been clustered or merged into the same node (with justification).*_	***3–4***

Information sources	7	Describe all information sources (e.g., databases with dates of coverage, contact with study authors to identify additional studies) in the search and date last searched.	3
Search	8	Present full electronic search strategy for at least one database, including any limits used, such that it could be repeated.	3
Study selection	9	State the process for selecting studies (i.e., screening, eligibility, included in systematic review, and, if applicable, included in the meta-analysis).	3–4
Data collection process	10	Describe method of data extraction from reports (e.g., piloted forms, independently, in duplicate) and any processes for obtaining and confirming data from investigators.	3
Data items	11	List and define all variables for which data were sought (e.g., PICOS, funding sources) and any assumptions and simplifications made.	4
**Geometry of the network**	**S1**	Describe methods used to explore the geometry of the treatment network under study and potential biases related to it. This should include how the evidence base has been graphically summarized for presentation, and what characteristics were compiled and used to describe the evidence base to readers.	***6***
Risk of bias within individual studies	12	Describe methods used for assessing risk of bias of individual studies (including specification of whether this was done at the study or outcome level), and how this information is to be used in any data synthesis.	6
Summary measures	13	State the principal summary measures (e.g., risk ratio, difference in means). *Also describe the use of additional summary measures assessed, such as treatment rankings and surface under the cumulative ranking curve (SUCRA) values, as well as modified approaches used to present summary findings from meta-analyses.*	7
Planned methods of analysis	14	Describe the methods of handling data and combining results of studies for each network meta-analysis. This should include, but not be limited to:<br>• *Handling of multi-arm trials;*<br>• *Selection of variance structure;*<br>• *Selection of prior distributions in Bayesian analyses; and*<br>• *Assessment of model fit.*	3
**Assessment of Inconsistency**	**S2**	Describe the statistical methods used to evaluate the agreement of direct and indirect evidence in the treatment network(s) studied. Describe efforts taken to address its presence when found.	**6–7**
Risk of bias across studies	15	Specify any assessment of risk of bias that may affect the cumulative evidence (e.g., publication bias, selective reporting within studies).	**3, 7, 9**
Additional analyses	16	Describe methods of additional analyses if done, indicating which were pre-specified. This may include, but not be limited to, the following:<br>• Sensitivity or subgroup analyses;	**3, 7**

		• Meta-regression analyses;<br>• *Alternative formulations of the treatment network; and*<br>• *Use of alternative prior distributions for Bayesian analyses (if applicable).*	
**RESULTS†**			
Study selection	17	Give numbers of studies screened, assessed for eligibility, and included in the review, with reasons for exclusions at each stage, ideally with a flow diagram.	4–5
**Presentation of network structure**	**S3**	Provide a network graph of the included studies to enable visualization of the geometry of the treatment network.	***6***
**Summary of network geometry**	**S4**	Provide a brief overview of characteristics of the treatment network. This may include commentary on the abundance of trials and randomized patients for the different interventions and pairwise comparisons in the network, gaps of evidence in the treatment network, and potential biases reflected by the network structure.	***6***
Study characteristics	18	For each study, present characteristics for which data were extracted (e.g., study size, PICOS, follow-up period) and provide the citations.	4–5,(Table 1)
Risk of bias within studies	19	Present data on risk of bias of each study and, if available, any outcome level assessment.	6 ,(Figure 2)
Results of individual studies	20	For all outcomes considered (benefits or harms), present, for each study: 1) simple summary data for each intervention group, and 2) effect estimates and confidence intervals. *Modified approaches may be needed to deal with information from larger networks.*	6–8
Synthesis of results	21	Present results of each meta-analysis done, including confidence/credible intervals. *In larger networks, authors may focus on comparisons versus a particular comparator (e.g. placebo or standard care), with full findings presented in an appendix. League tables and forest plots may be considered to summarize pairwise comparisons.* If additional summary measures were explored (such as treatment rankings), these should also be presented.	***3–7***
**Exploration for inconsistency**	**S5**	Describe results from investigations of inconsistency. This may include such information as measures of model fit to compare consistency and inconsistency models, *P* values from statistical tests, or summary of inconsistency estimates from different parts of the treatment network.	***6***
Risk of bias across studies	22	Present results of any assessment of risk of bias across studies for the evidence base being studied.	7 ,(Figure 6)
Results of additional analyses	23	Give results of additional analyses, if done (e.g., sensitivity or subgroup analyses, meta-regression analyses*, alternative network geometries studied, alternative choice of prior distributions for Bayesian analyses,* and so forth).	***7***

**DISCUSSION**			
Summary of evidence	24	Summarize the main findings, including the strength of evidence for each main outcome; consider their relevance to key groups (e.g., healthcare providers, users, and policy-makers).	7–9
Limitations	25	Discuss limitations at study and outcome level (e.g., risk of bias), and at review level (e.g., incomplete retrieval of identified research, reporting bias). *Comment on the validity of the assumptions, such as transitivity and consistency. Comment on any concerns regarding network geometry (e.g., avoidance of certain comparisons).*	9
Conclusions	26	Provide a general interpretation of the results in the context of other evidence, and implications for future research.	10
**FUNDING**			
Funding	27	Describe sources of funding for the systematic review and other support (e.g., supply of data); role of funders for the systematic review. This should also include information regarding whether funding has been received from manufacturers of treatments in the network and/or whether some of the authors are content experts with professional conflicts of interest that could affect use of treatments in the network.	***10***

PICOS = population, intervention, comparators, outcomes, study design.

* Text in italics indicateS wording specific to reporting of network meta-analyses that has been added to guidance from the PRISMA statement.

† Authors may wish to plan for use of appendices to present all relevant information in full detail for items in this section.

Table S2 Search strategy in PubMed

Search	Query
#5	Search: (#1) AND (#2) AND (#3) AND (#4)
#4	Search: ("Randomized Controlled Trial"[Publication Type] OR "Randomized trial" OR "RCT" OR "Controlled trial")
#3	Search: ("Depression"[MeSH] OR "Depressive symptoms" OR "Depressive symptom" OR "Symptom, depressive" OR "Emotional depression" OR "Depression, emotional")
#2	Search: ("Combined Modality Therapy"[MeSH] OR "Therapy, combined modality" OR "Combined modality therapies" OR "Modality therapies, combined" OR "Modality therapy, combined" OR "Therapies, combined modality" OR "Multimodal treatment" OR "Multimodal treatments" OR "Treatment, multimodal" OR "Treatments, multimodal")
#1	Search: ("Exercise"[MeSH] OR "Physical exercise" OR "Physical exercises" OR "Physical activity" OR "Activities, physical" OR "Activity, physical" OR "Physical activities" OR "Aerobic exercise" OR "Aerobic exercises" OR "Exercises, aerobic" OR "Isometric exercise" OR "Isometric exercises" OR "Exercises, isometric" OR "Acute exercise" OR "Acute exercises" OR "Exercise, acute" OR "Exercises, acute" OR "Exercise training" OR "Exercise trainings" OR "Training, exercise" OR "Trainings, exercise")

Table S3 Search strategy in Web of Science

Search	Query
#5	Search: (#1) AND (#2) AND (#3) AND (#4)
#4	Search: TS=("Randomized Controlled Trial" OR "Randomized trial" OR "RCT" OR "Controlled trial")
#3	Search: TS=("Depression" OR "Depressive Symptoms" OR "Depressive Symptom" OR "Symptom, Depressive" OR "Emotional Depression" OR "Depression, Emotional")
#2	Search: TS=("Combined Modality Therapy" OR "Therapy, Combined Modality" OR "Combined Modality Therapies" OR "Modality Therapies, Combined" OR "Modality Therapy, Combined" OR "Therapies, Combined Modality" OR "Multimodal Treatment" OR "Multimodal Treatments" OR "Treatment, Multimodal" OR "Treatments, Multimodal")
#1	Search: TS=("Exercise" OR "Physical Exercise" OR "Physical Exercises" OR "Physical Activity" OR "Activities, Physical" OR "Activity, Physical" OR "Physical Activities" OR "Aerobic Exercise" OR "Aerobic Exercises" OR "Exercises, Aerobic" OR "Isometric Exercise" OR "Isometric Exercises" OR "Exercises, Isometric" OR "Acute Exercise" OR "Acute Exercises" OR "Exercise, Acute" OR "Exercises, Acute" OR "Exercise Training" OR "Exercise Trainings" OR "Training, Exercise" OR "Trainings, Exercise")

Table S4 Search strategy in Cochrane Library

Search	Query
#5	Search: (#1) AND (#2) AND (#3) AND (#4)
#4	Search: ("Randomized controlled trial" OR "Randomized trial" OR "RCT" OR "Controlled trial")
#3	Search: ("Depression" OR "Depressive symptoms" OR "Depressive symptom" OR "Symptom, depressive" OR "Emotional depression" OR "Depression, emotional")
#2	Search: ("Combined modality therapy" OR "Therapy, combined modality" OR "Combined modality therapies" OR "Modality therapies, combined" OR "Modality therapy, combined" OR "Therapies, combined modality" OR "Multimodal treatment" OR "Multimodal treatments" OR "Treatment, multimodal" OR "Treatments, multimodal")
#1	Search: ("Exercise" OR "Physical exercise" OR "Physical exercises" OR "Physical activity" OR "Activities, physical" OR "Activity, physical" OR "Physical activities" OR "Aerobic exercise" OR "Aerobic exercises" OR "Exercises, aerobic" OR "Isometric exercise" OR "Isometric exercises" OR "Exercises, isometric" OR "Acute exercise" OR "Acute exercises" OR "Exercise, acute" OR "Exercises, acute" OR "Exercise training" OR "Exercise trainings" OR "Training, exercise" OR "Trainings, exercise")

Table S5 Search strategy in Embase

Search	Query
#5	Search: (#1) AND (#2) AND (#3) AND (#4)
#4	Search: ('Randomized Controlled Trial' OR 'randomized trial' OR 'RCT' OR 'controlled trial')
#3	Search: ('Depression' OR 'Depressive Symptoms' OR 'Depressive Symptom' OR 'Symptom, Depressive' OR 'Emotional Depression' OR 'Depression, Emotional')
#2	Search: ('Combined Modality Therapy' OR 'Therapy, Combined Modality' OR 'Combined Modality Therapies' OR 'Modality Therapies, Combined' OR 'Modality Therapy, Combined' OR 'Therapies, Combined Modality' OR 'Multimodal Treatment' OR 'Multimodal Treatments' OR 'Treatment, Multimodal' OR 'Treatments, Multimodal')
#1	Search: ('Exercise' OR 'Physical Exercise' OR 'Physical Exercises' OR 'Physical Activity' OR 'Activities, Physical' OR 'Activity, Physical' OR 'Physical Activities' OR 'Aerobic Exercise' OR 'Aerobic Exercises' OR 'Exercises, Aerobic' OR 'Isometric Exercise' OR 'Isometric Exercises' OR 'Exercises, Isometric' OR 'Acute Exercise' OR 'Acute Exercises' OR 'Exercise, Acute' OR 'Exercises, Acute' OR 'Exercise Training' OR 'Exercise Trainings' OR 'Training, Exercise' OR 'Trainings, Exercise')

Table S6 Search strategy in Scopus

Search	Query
#5	Search: (#1) AND (#2) AND (#3) AND (#4)
#4	Search: (Randomized Controlled Trial OR randomized trial OR RCT OR controlled trial)
#3	Search: (Depression OR Depressive Symptoms OR Depressive Symptom OR Symptom, Depressive OR Emotional Depression OR Depression, Emotional)
#2	Search: (Combined Modality Therapy OR Therapy, Combined Modality OR Combined Modality Therapies OR Modality Therapies, Combined OR Modality Therapy, Combined OR Therapies, Combined Modality OR Multimodal Treatment OR Multimodal Treatments OR Treatment, Multimodal OR Treatments, Multimodal)
#1	Search: (Exercise OR Physical Exercise OR Physical Exercises OR Physical Activity OR Activities, Physical OR Activity, Physical OR Physical Activities OR Aerobic Exercise OR Aerobic Exercises OR Exercises, Aerobic OR Isometric Exercise OR Isometric Exercises OR Exercises, Isometric OR Acute Exercise OR Acute Exercises OR Exercise, Acute OR Exercises, Acute OR Exercise Training OR Exercise Trainings OR Training, Exercise OR Trainings, Exercise)

Table S7 Search strategy in CNKI

Search	Query
#4	主题: (#1) AND 主题: (#2) AND 主题: (#3)
#3	主题: "抑郁症+抑郁症患者"
#2	主题: "联合+联合治疗+联合用药+联合使用+联合应用"
#1	主题: "运动+有氧运动"

Table S8 Search strategy in Wanfang database

Search	Query
#4	主题: (#1) AND 主题: (#2) AND 主题: (#3)
#3	主题: (抑郁症 OR 抑郁症患者)
#2	主题: (联合 OR 联合治疗 OR 联合用药 OR 联合使用 OR 联合应用)
#1	主题: (运动 OR 有氧运动)

Table S9 Search strategy in China Biomedical Literature Database

Search	Query
#4	(#1) AND (#2) AND (#3)
#3	("抑郁症"[全部字段:智能] OR "抑郁症患者"[全部字段:智能])
#2	("联合"[全部字段:智能] OR "联合治疗"[全部字段:智能] OR "联合用药"[全部字段:智能] OR "联合使用"[全部字段:智能] OR "联合应用"[全部字段:智能])
#1	("运动"[全部字段:智能] OR "有氧运动"[全部字段:智能])

Table S10 Inclusion and Exclusion Criteria

Term	Inclusion	Exclusion
Population	- Patients of all ages with confirmed depression diagnosis (DSM,ICD,or CCMD)<br>- Patients exceeding clinical thresholds on validated depression scales (e.g.,HAMD≥8,BDI≥14)<br>- Comorbid conditions permitted	- No formal depression diagnosis OR scales below clinical threshold<br>- Studies not specifically targeting depression (e.g., other primary diagnoses)
Intervention	- Experimental Group:<br>- Adjunctive continuous aerobic exercise<br>- Combined with conventional treatment (meds/psychotherapy/physical therapy)	-Use of only a single treatment intervention
Comparison	Control Group:<br>- Conventional treatment only (medication, psychotherapy, physical therapy, OR single-mode exercise therapy)	- No conventional treatment comparator<br>- Different exercise modalities as control
Outcome	- Depressive symptom severity changes measured by:<br>• Hamilton Depression Scale (HAMD)<br>• Beck Depression Inventory (BDI)<br>• Self-rating Depression Scale (SDS)	- Non-validated assessment tools<br>- Incomplete outcome data<br>- Physiological/biomarker outcomes without clinical symptom reporting
Study	- RCTs (individual design, cluster design, or the first half of crossover)	Non-RCTs

Table S11 SUCRA cumulative probability ranking (based on HAMD)

RANK	SUCRA	PrBest	MeanRank
ECT+AE	97.6	86.4	1.2
rTMS+AE	75.5	7	3.2
TCM+AE	68.4	4	3.8
SSRI+AE	67.3	1.3	3.9
CBT+AE	55.6	0.8	5
PT	41.2	0	6.3
EX	36.5	0	6.7
CBT	22.1	0.4	8
TCM	22	0	8
CT	13.7	0	8.8

Table S12 SUCRA cumulative probability ranking (based on BDI)

RANK	SUCRA	PrBest	MeanRank
SSRI+AE	88.8	48.6	1.7
ECT+AE	78.1	38	2.3
CBT+AE	75	13.1	2.5
EX	33.1	0	5
CBT	30.9	0	5.1
CT	26.6	0	5.4
PT	17.3	0.3	6

Table S13 SUCRA cumulative probability ranking (based on SDS)

RANK	SUCRA	PrBest	MeanRank
TCM+AE	83.6	50.8	1.5
CBT+AE	83.1	49.2	1.5
CT	29.4	0	3.1
CBT	4	0	3.9

Table S14 Evaluation of heterogeneity

Primary outcome	τ^2	Q	df	P	I^2	Heterogeneity assessment
HAMD	4.4702	9345.45	32	0.0001	99.7	High
BDI	12.5791	1904.97	18	0.0001	99.1	High
SDS	3.7506	216.01	5	0.0001	97.7	High

Table S15 Outcome of Network Meta-Regression (median and 95%Crl)

Primary outcome	Age	Publication year	Diagnostic tool	Baseline depresion statue	Frequence	Time(minutes)	Duration(weeks)
HAMD	-6.97 (-47.41 - 3.82)	-0.48 (-2.47 - 8.56)	1.44 (-29.10 - 5.94)	0.48 (-2.62 - 24.65)	0.03 (-15.55 - 3.86)	-1.08 (-3.98 - 1.50)	0.23 (-1.93 - 3.17)
BDI	-7.34 (-44.23 - 23.17)	-0.85 (-75.91 - 117.68)	-3.55 (-232.30 - 41.33)	1.80 (-48.96 - 195.90)	-2.88 (-286.15 - 108.40)	-0.09 (-45.10 - 78.24)	0.25 (-45.98 - 45.72)
SDS	0.51 (-11.04 - 15.05)	0.32 (-7.11 - 6.65)	0.43 (-11.23 - 11.36)	-0.23 (-57.46 - 35.54)	-0.99 (-18.85 - 17.06)	-0.36 (-22.01 - 23.00)	-0.524 (-11.21 - 10.36)

Table S16 The Overall Certainty of Evidence (CINeMA，Direct)

Comparison	Number of studies	Within-study bias	Reporting bias	Indirectness	Imprecision	Heterogeneity	Incoherence	Confidence rating	Reason(s) for downgrading
HAMD									
CT:EX	1	No concerns	Low risk	No concerns	Major concerns	No concerns	No concerns	Low	[Imprecision]
CT:TCM+AE	2	Some concerns	Low risk	No concerns	No concerns	Some concerns	No concerns	Moderate	[Within-study bias, Heterogeneity]
CT:CBT+AE	2	No concerns	Low risk	No concerns	No concerns	Major concerns	No concerns	Low	[Heterogeneity]
CT:SSRI+AE	15	No concerns	Low risk	No concerns	No concerns	No concerns	No concerns	High	
BDI									
CBT:CT	2	No concerns	Low risk	No concerns	Major concerns	No concerns	No concerns	Low	[Imprecision]
CBT+AE:CT	2	No concerns	Low risk	No concerns	No concerns	Major concerns	No concerns	Low	[Heterogeneity]
CT:EX	4	No concerns	Low risk	No concerns	Major concerns	No concerns	No concerns	Low	[Imprecision]
CT:SSRI+AE	6	No concerns	Low risk	No concerns	No concerns	Major concerns	No concerns	Low	[Heterogeneity]
SDS									
CBT+AE:CT	2	No concerns	Low risk	No concerns	No concerns	Major concerns	Major concerns	Very low	[Heterogeneity, Incoherence]
CT:TCM+AE	2	Some concerns	Low risk	No concerns	No concerns	Major concerns	Major concerns	Very low	[Heterogeneity, Incoherence]

Table S17 The Overall Certainty of Evidence (CINeMA，Indirect)

Comparison	Number of studies	Within-study bias	Reporting bias	Indirectness	Imprecision	Heterogeneity	Incoherence	Confidence rating	Reason(s) for downgrading
HAMD									
CT:CBT	0	No concerns	Low risk	No concerns	Major concerns	No concerns	No concerns	Low	[Imprecision]
CT:ECT+AE	0	No concerns	Low risk	No concerns	No concerns	No concerns	No concerns	High	
CT:PT	0	No concerns	Low risk	No concerns	Major concerns	No concerns	No concerns	Low	[Imprecision]
CT:TCM	0	Some concerns	Low risk	No concerns	Major concerns	No concerns	No concerns	Low	[Within-study bias, Imprecision]
CT:rTMS+AE	0	No concerns	Low risk	No concerns	No concerns	Major concerns	No concerns	Low	[Heterogeneity]
BDI									
CT:ECT+AE	0	No concerns	Low risk	No concerns	Major concerns	No concerns	No concerns	Low	[Imprecision]
CT:PT	0	No concerns	Low risk	No concerns	Major concerns	No concerns	No concerns	Low	[Imprecision]
SDS									
CBT:CT	0	No concerns	Low risk	No concerns	No concerns	Major concerns	Major concerns	Very low	[Heterogeneity, Incoherence]

Reason for Downgrading

Based on the recommendations from the CINeMA online documentation (https://cinema.ispm.unibe.ch/). the need for downgrading in each domain was assessed according to the criteria outlined below. The methodology referenced the CINeMA guidance (1).

Within-study bias

Based on the risk of bias in each domain (SupplementaryFigure), each study was judged as "Low risk", "Some concerns", or "High risk". According to the risk of bias assessment tool recommended by Cochrane Handbook 5.1.0, the

judgements were made using RevMan 5.4 software following the criteria for randomized controlled trials(including random sequence generation, allocation concealment, blinding of participants and personnel, blinding of outcome assessment, incomplete outcome data, selective reporting, and other sources of bias) . When all items were assessed as "Low risk", the study was rated as "Low risk";when some items were "Low risk", it was categorized as "Some concerns";if none was judged as "Low risk", it was deemed "High risk" .

Reporting bias

The search strategy was comprehensive, with no language restrictions applied; both Chinese and English publications were included in the screening process.The final pooled results remained unaffected even if any unpublished small-scale trials were missed during the search. Publication bias was assessed using Supplementary Figure S14, which demonstrated no signs of asymmetry in the trim-and-fill funnel plots for most combination therapy comparisons. Based on the funnel plot analysis, we downgraded the evidence quality for the relevant comparisons.

Indirectness

Indirectness refers to the relevance of the included studies to the research question . Study populations, interventions, outcomes, and study settings might not be representative of the settings, populations, or outcomes about which reviewers want to make inferences. We addressed this issue by strictly adhering to the inclusion and exclusion criteria(limited to subjects with depression scores above the threshold and without excluding based on comorbidity)therefore, no downgrading was required in this domain.

Imprecision

The outcome measures (HAMD, BDI, SDS) in this network meta-analysis were continuous variables. Given the use of different rating scales or units across studies, mean difference (MD) was selected as the effect measure for continuous variables, calculated based on change scores (final score minus baseline score). We established the effectiveness thresholds for various interventions combined with aerobic exercise at 0.8 or 1.25 (1).The judgment rules were as follows:

1. If the confidence interval crosses the null line and extends into the range favoring the opposite intervention, it is judged as "Major concerns".
2. If the confidence interval only contains null effects (or additionally includes clinically important values favoring the same direction as the point estimate), it is judged as "Some concerns".
3. If the confidence interval solely contains clinically important values favoring the same direction as the point estimate, it is judged as "No concerns".
4. If the confidence interval lies entirely between two clinically important thresholds, it is similarly judged as "No concerns".

Heterogeneity

In the assessment of heterogeneity, we employed the same criteria as the clinically important thresholds described earlier. Following the automated recommendations of CINeMA, the generation of conclusions was based on the alignment between confidence intervals and prediction intervals. Specifically, CINeMA makes its judgments by evaluating the interval alignment for the following two values:

5. The null effect value (MD=0)
6. And the clinically important effect value in the opposite direction to the point estimate.

Incoherence

In the assessment of Incoherence, we employed CINeMA to compare direct and indirect evidence, integrating the results of the following tests:

Global test based on a random-effects design-by-treatment interaction model。

X^2 statistic: 1.939 (1 degrees of freedom), P value: 0.164。

Design-by-treatment interaction test and the method of Separating Indirect from Direct Evidence (SIDE). The specific decision rules were as follows: For estimates informed by both direct and indirect evidence: SIDE test p-value > 0.10 → "No concerns". SIDE test p-value < 0.10 → Risk level judged based on the agreement between the 95% confidence intervals of the direct and indirect estimates within the range of clinically important effects. For estimates informed by only direct evidence or only indirect evidence: Judgment based on the p-value of the design-by-treatment interaction test. Special case: If no closed evidence loop exists, preventing the tests from being conducted → All comparisons are judged as "Major concerns".

Summarising judgments across the 6 domains

The final output table from CINeMA will present the concern level for each domain.We performed an overall judgment across domains, referencing the four levels of evidence quality (very low, low, moderate, high) from the GRADE framework,applying the following specific rules:

1. High: All domains are "No concerns"→Evidence quality remains "high".
2. Moderate: No "Major concerns" and ≤2 domains have "Some concerns"→Evidence quality is downgraded to "moderate".
3. Low: No "Major concerns" but ≥3 domains have "Some concerns"→Evidence quality is downgraded to "low".
4. Very low:≥2 domains have "Major concerns", OR 1 domain has "Major concerns" combined with ≥1 domain having "Some concerns"→Evidence quality is downgraded to "very low".

The 6 CINeMA domains should therefore be considered jointly rather than in isolation, avoiding downgrading the overall level of confidence more than once for related concerns.

Reference:

1． Nikolakopoulou Adriani,Higgins Julian P T,Papakonstantinou Theodoros,Chaimani Anna,Del Giovane Cinzia,Egger Matthias & Salanti Georgia.(2020).CINeMA: An approach for assessing confidence in the results of a network meta-analysis..PLoS medicine,17(4),e1003082.

2. *Supplementary Figure*

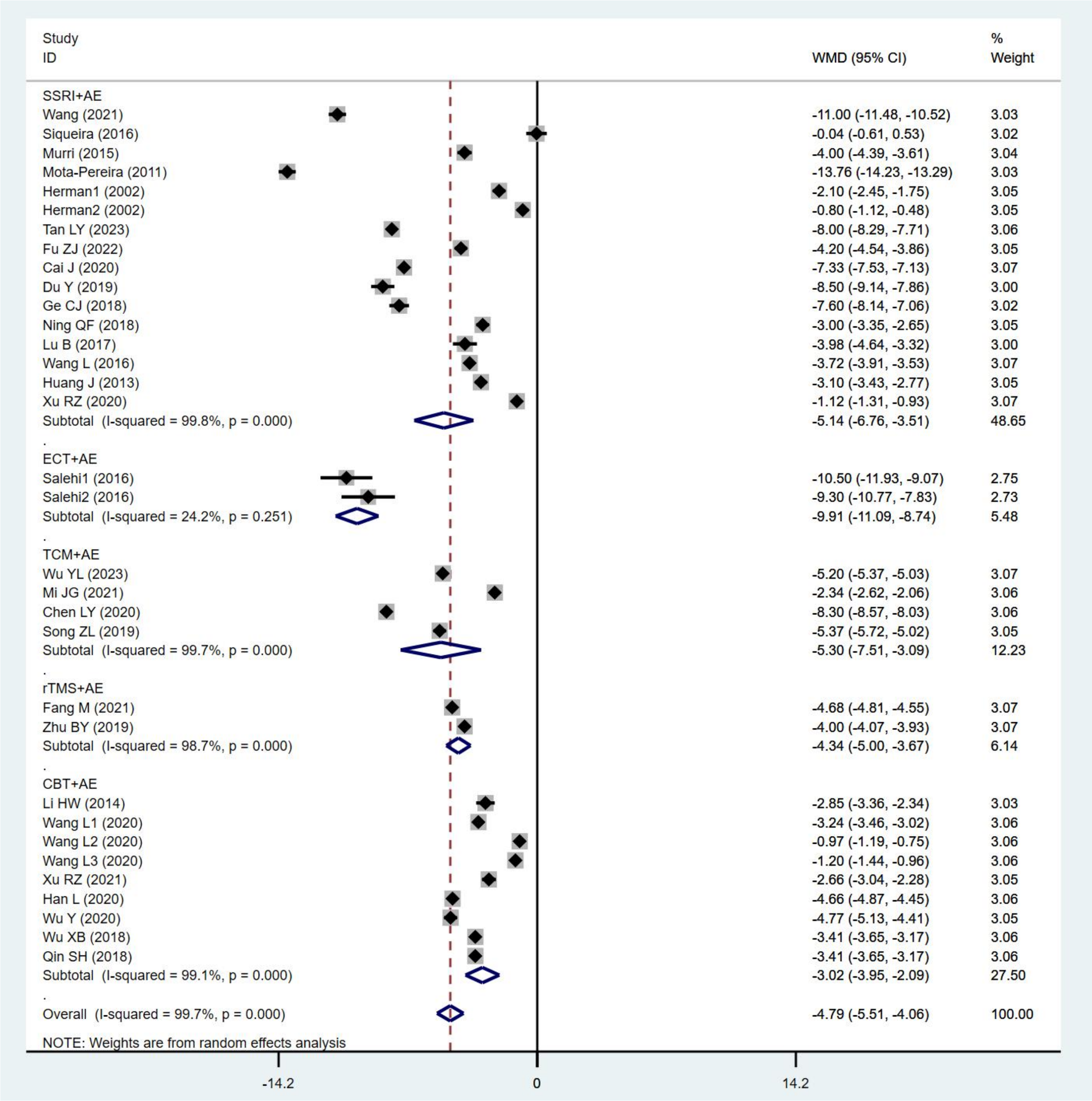

Figure S1 | Meta-analysis of the effect of different treatment measures combined with aerobic exercise on the intervention of depressed patients (based on HAMD)

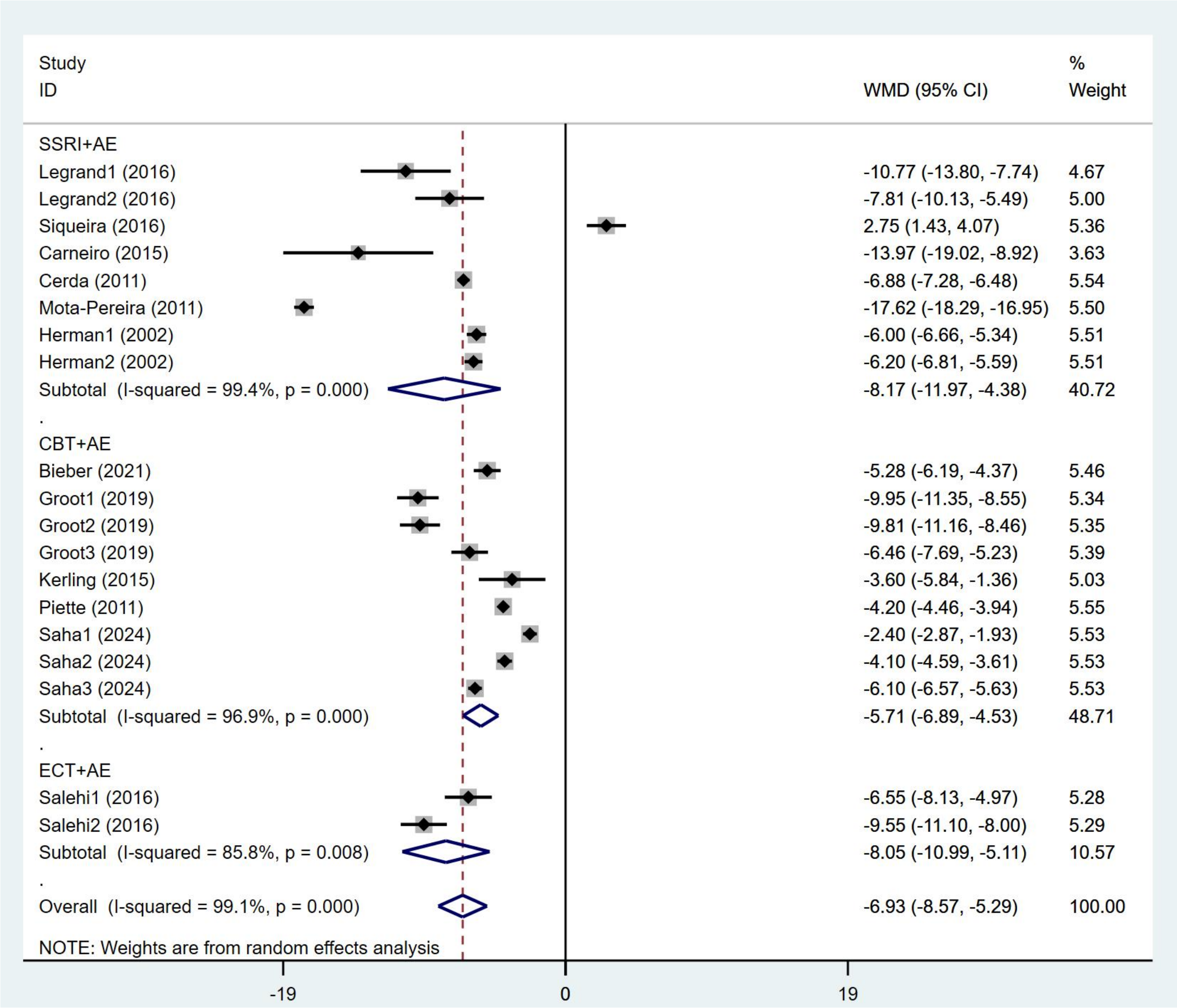

Figure S2 | Meta-analysis of the effect of different treatment measures combined with aerobic exercise on the intervention of depressed patients (based on BDI)

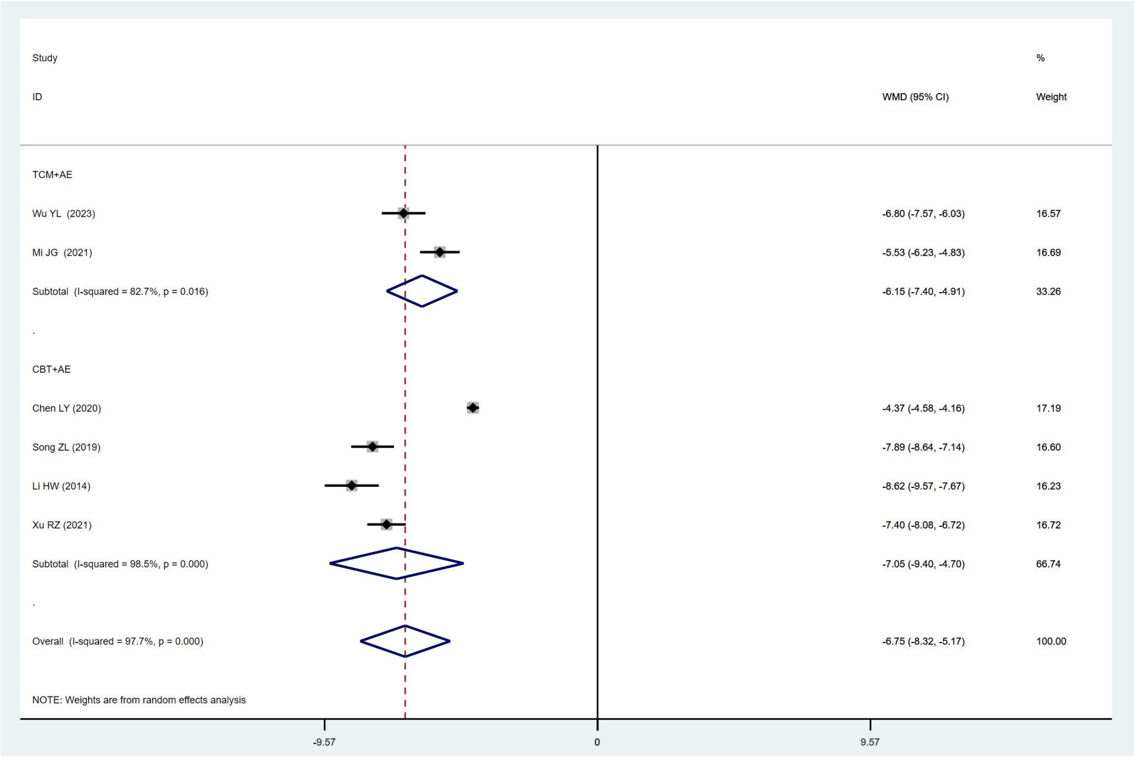

Figure S3 | Meta-analysis of the effect of different treatment measures combined with aerobic exercise on the intervention of depressed patients (based on SDS)

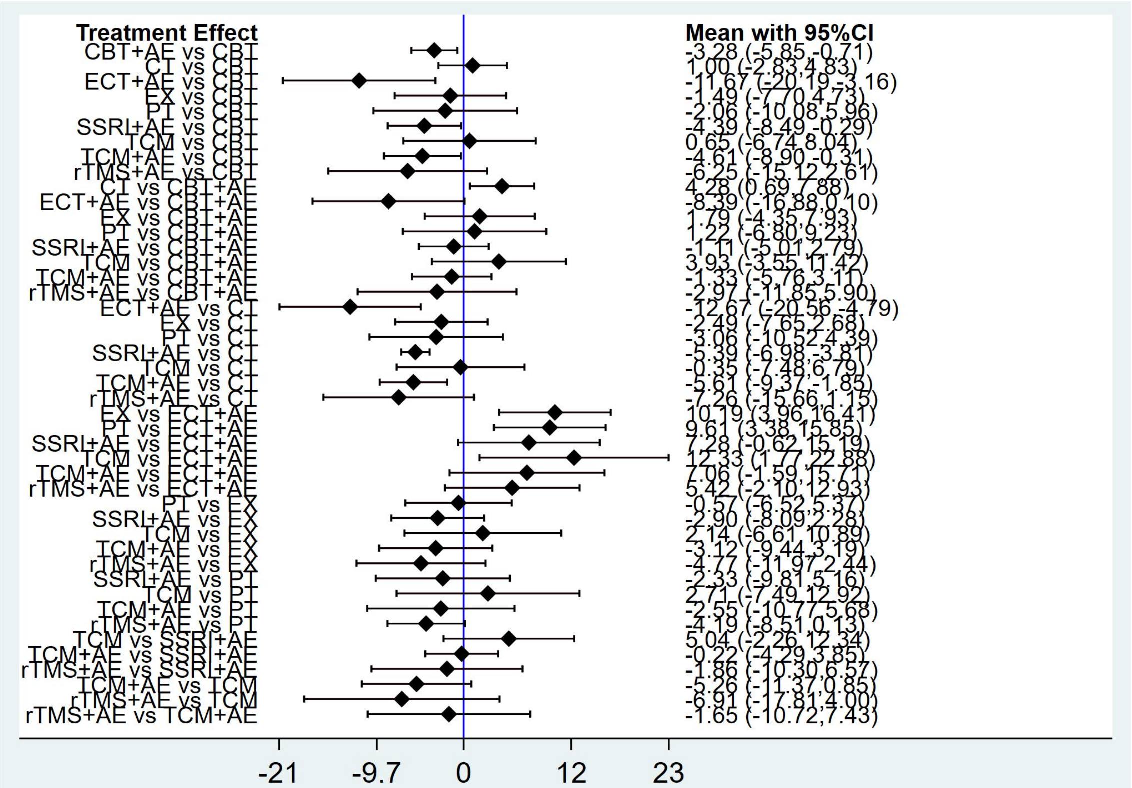

Figure S4 | Network meta-analysis interval plots for different treatment measures (HAMD-based)

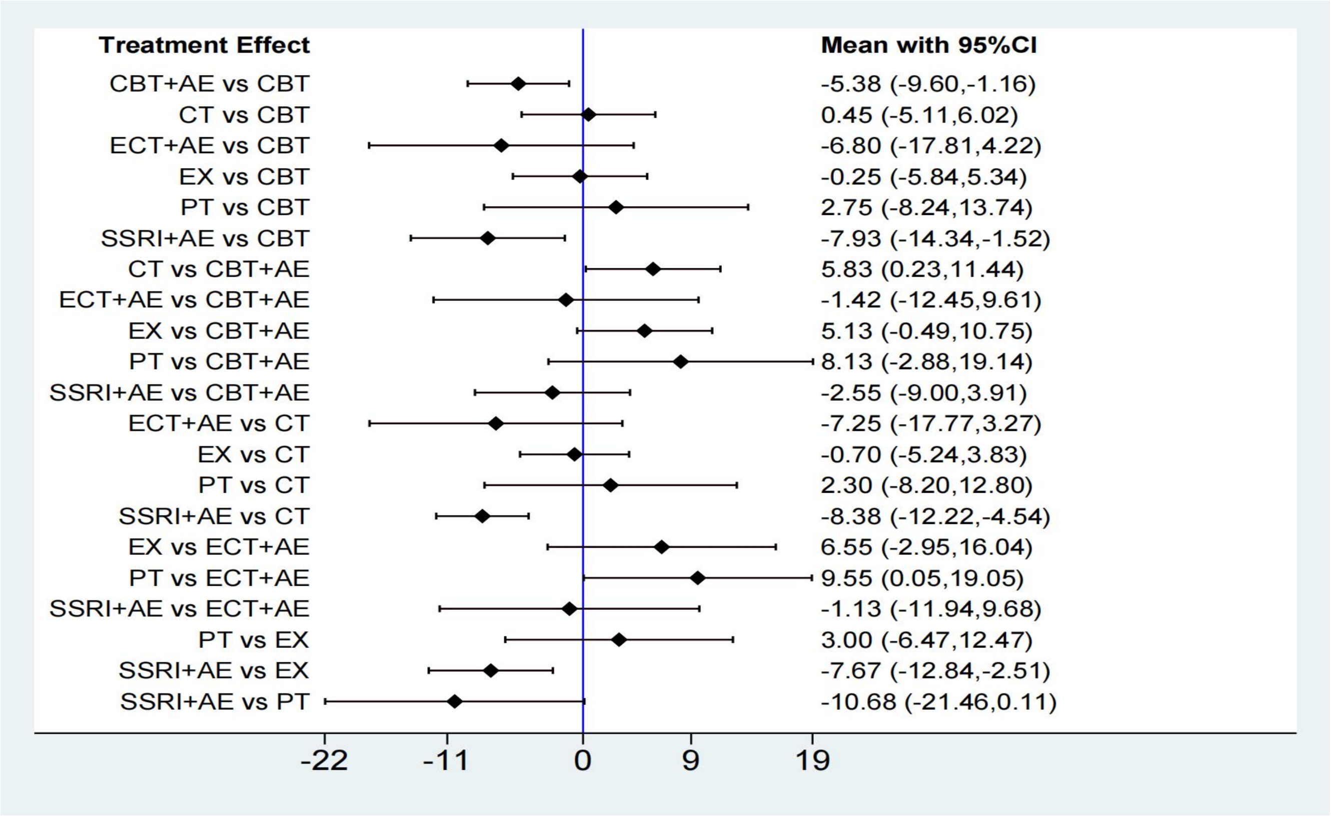

Figure S5 | Network meta-analysis interval plots for different treatment measures (BDI-based)

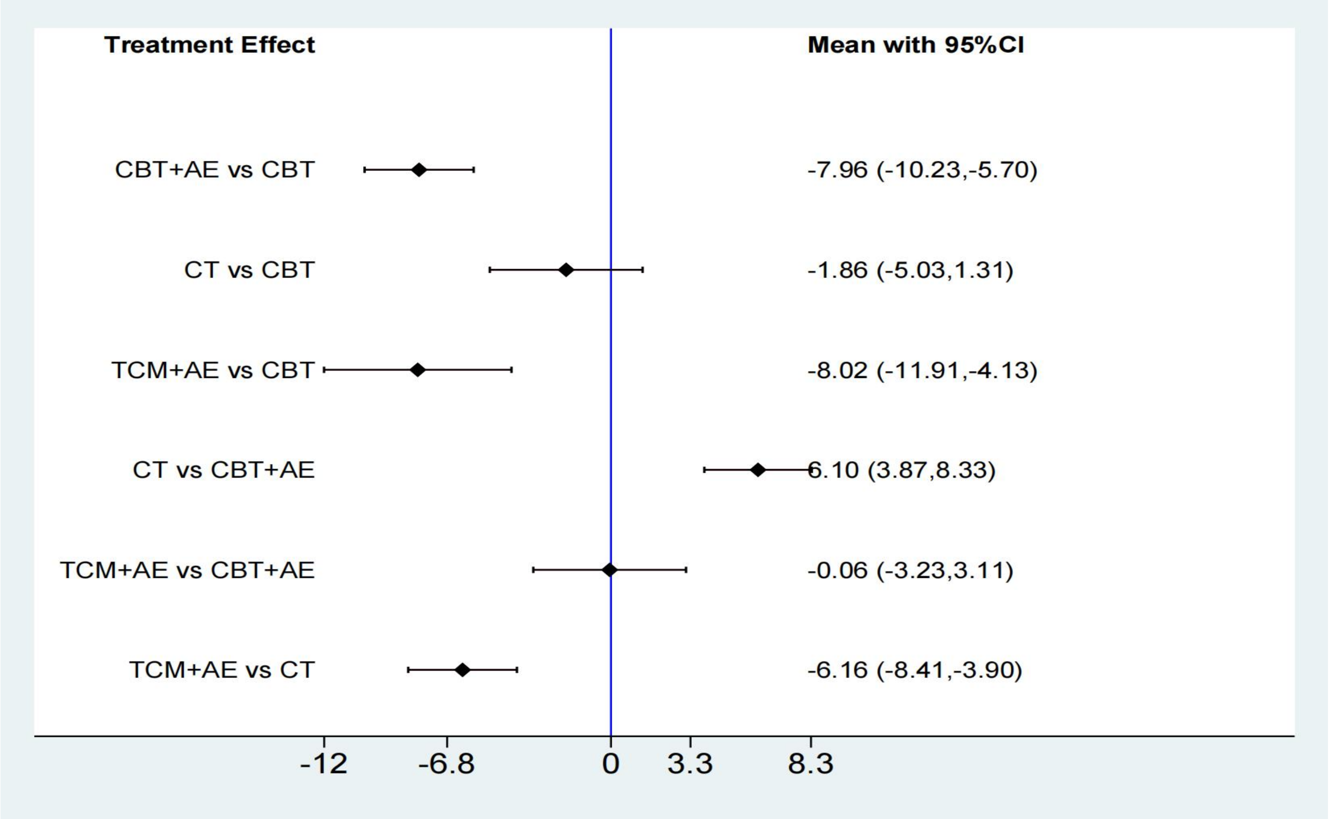

Figure S6 | Network meta-analysis interval plots for different treatment measures (SDS-based)

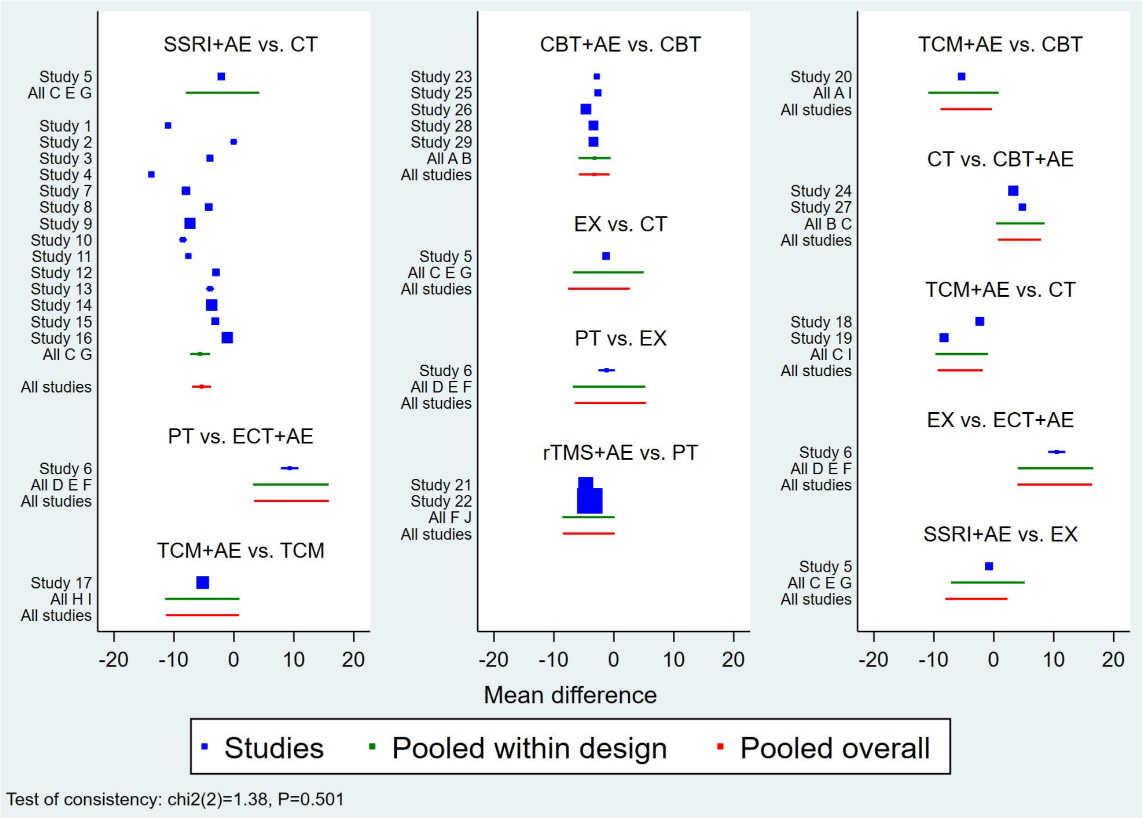

Figure S 7 | HAMD-based forest maps

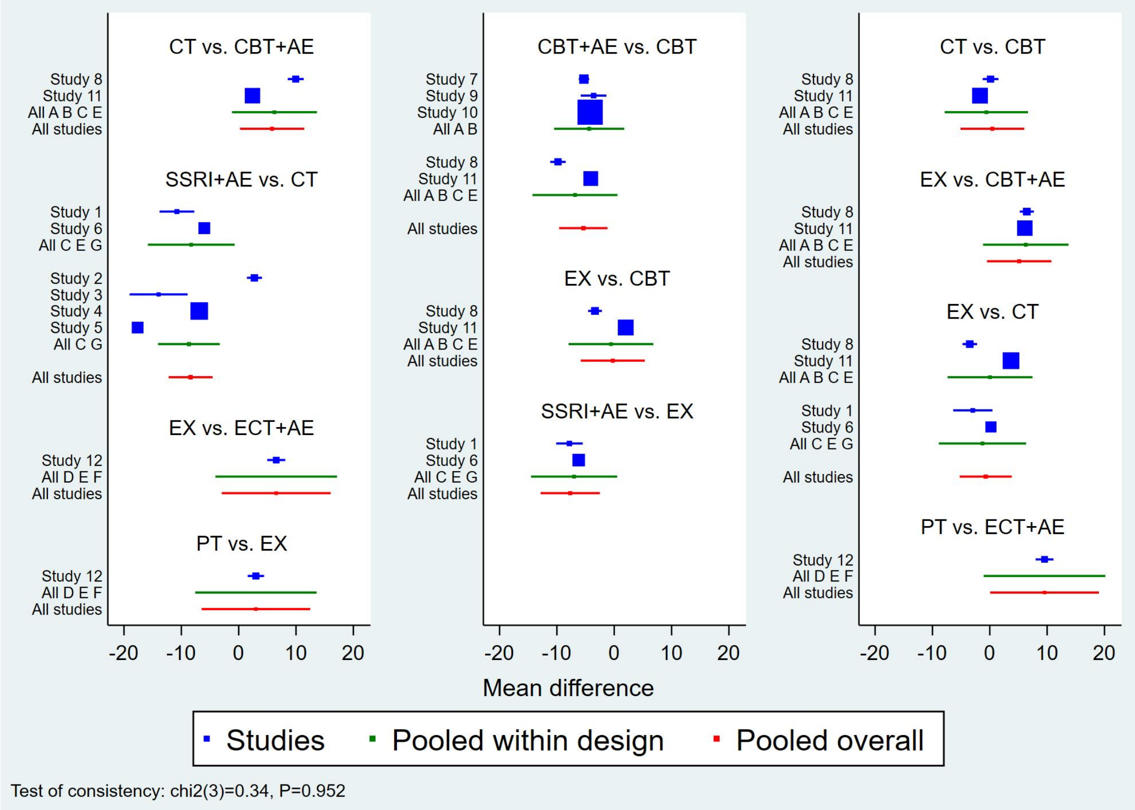

Figure S8 | BDI-based forest maps

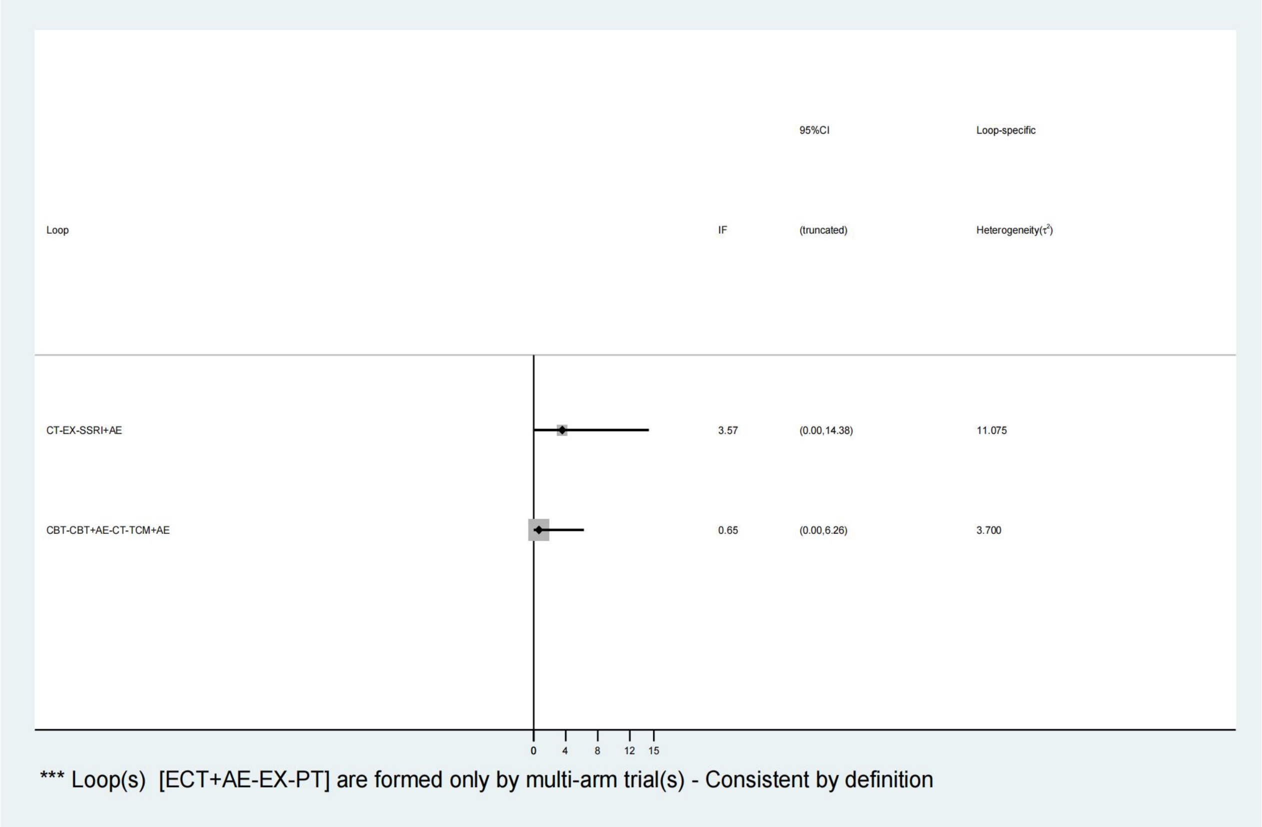

Figure S9 | Inconsistency test for closed loops (based on HAMD)

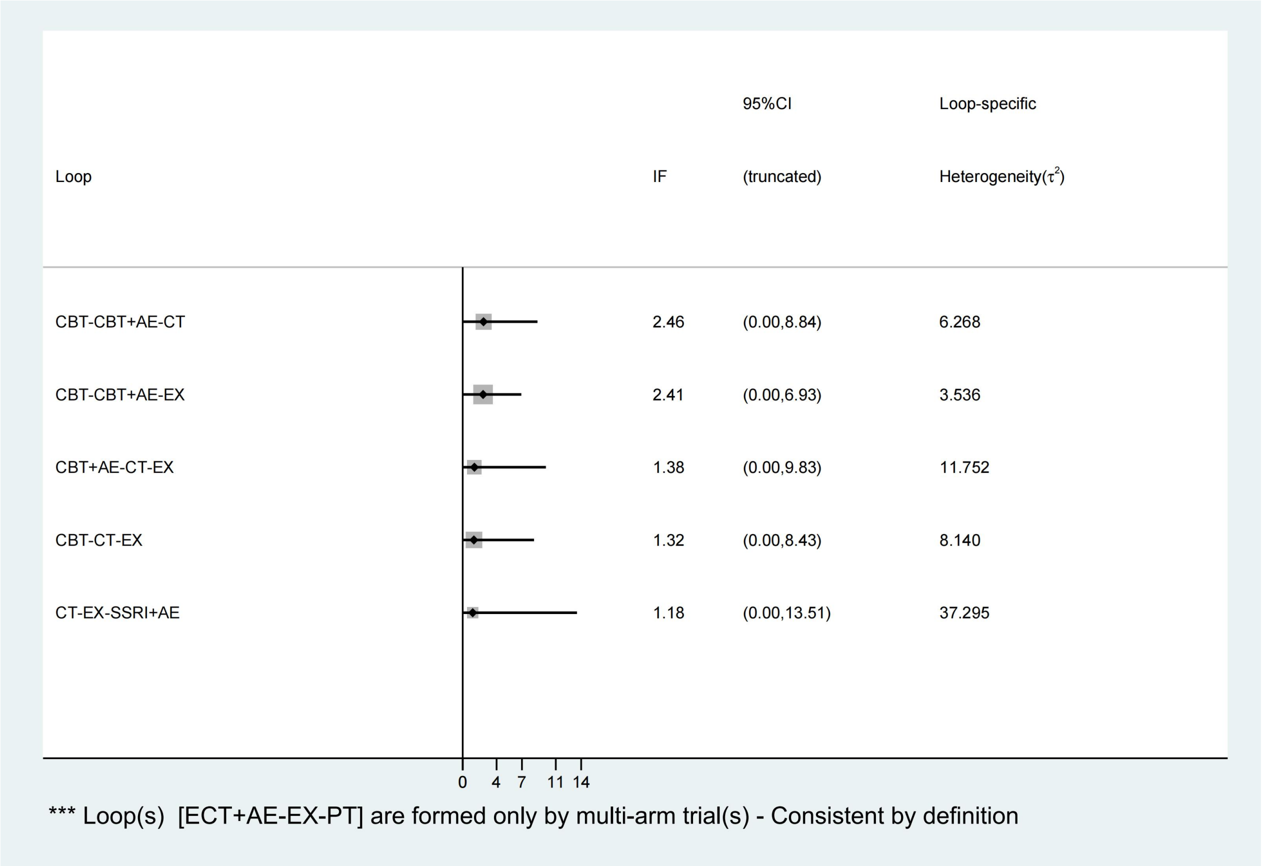

Figure S10 | Inconsistency test for closed loops (based on BDI)

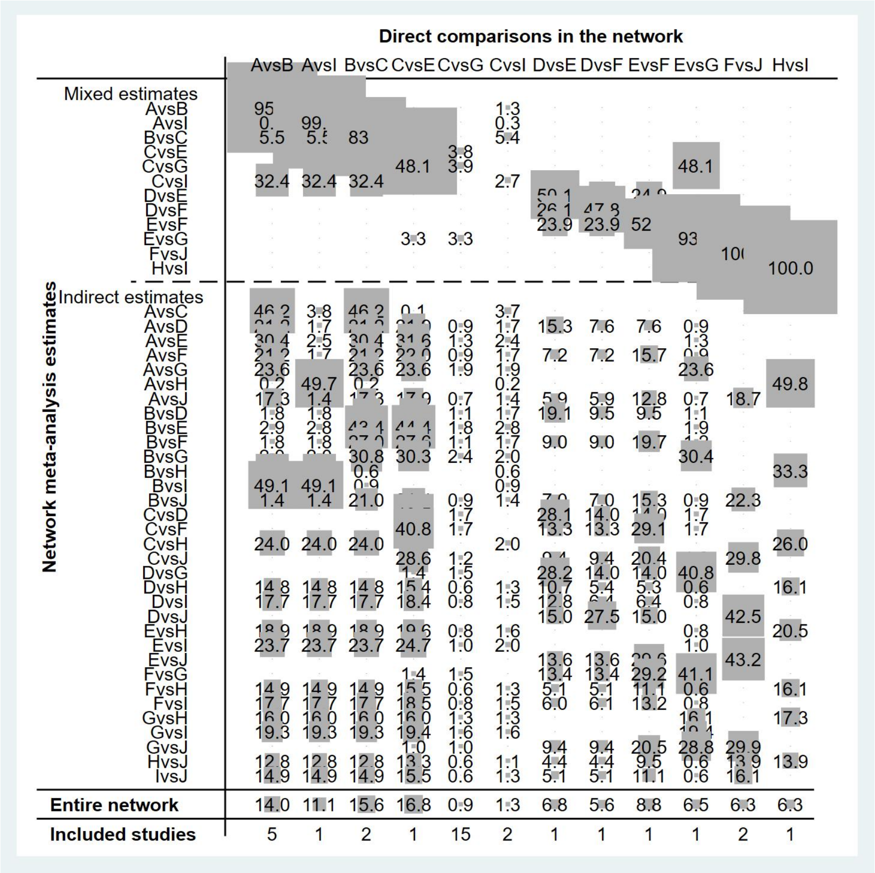

Figure S11 | Contribution of the HAMD scale

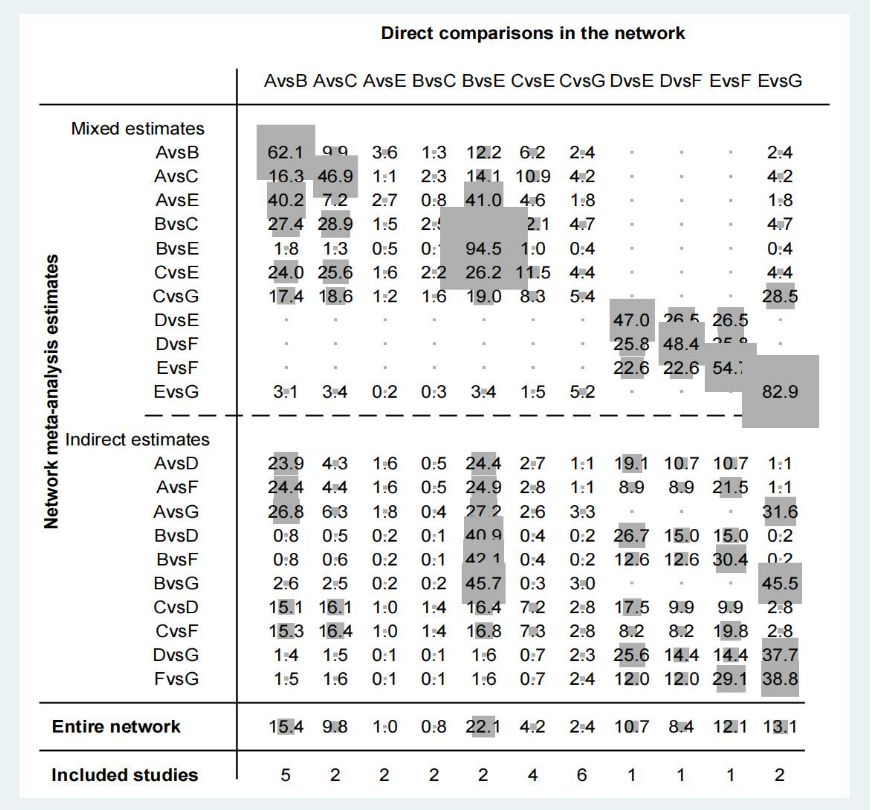

Figure S12 | Contribution of the BDI scale

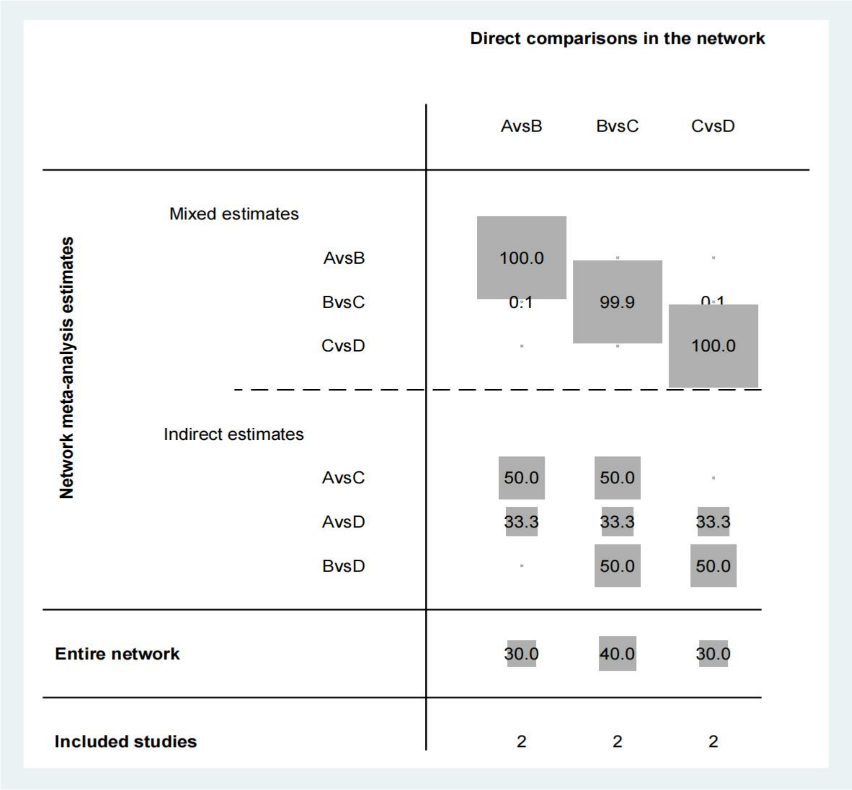

Figure S13 | Contribution of the SDS scale

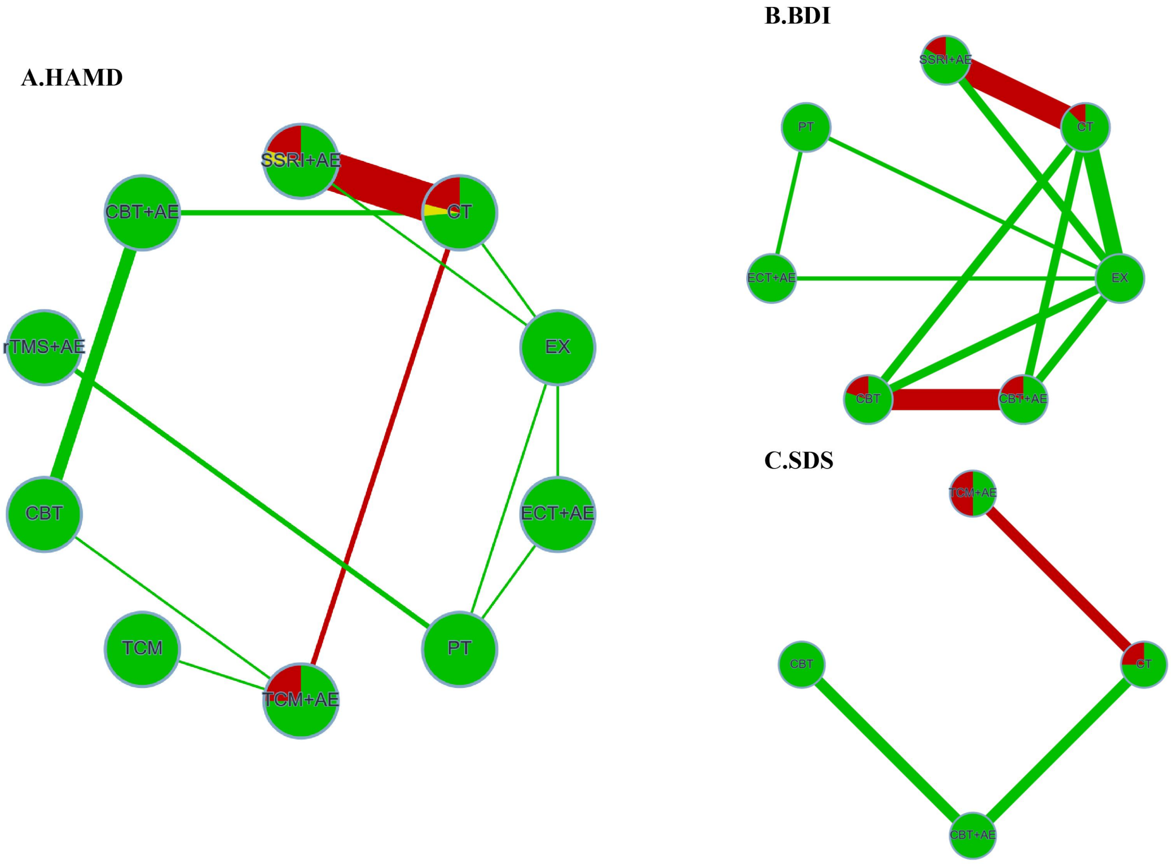

Figure S14 | Summary of limitations of included studies.
